# Expression and purification of the 5′-nucleotidase YitU from *Bacillus* species: its enzymatic properties and possible applications in biotechnology

**DOI:** 10.1007/s00253-020-10428-y

**Published:** 2020-02-10

**Authors:** Yuliya R. Yusupova, Victoria S. Skripnikova, Alexandr D. Kivero, Natalia P. Zakataeva

**Affiliations:** Ajinomoto-Genetika Research Institute, 1st Dorozhny Proezd, b.1-1, Moscow, 117545 Russia

**Keywords:** 5′-Nucleotidases (EC 3.1.3.5), Riboflavin producers, 5-Aminoimidazole-4-carboxamide ribonucleoside (AICAR) producers, *Bacillus subtilis*, *Bacillus amyloliquefaciens*

## Abstract

5’-Nucleotidases (EC 3.1.3.5) are enzymes that catalyze the hydrolytic dephosphorylation of 5′-ribonucleotides and 5′-deoxyribonucleotides to their corresponding nucleosides plus phosphate. In the present study, to search for new genes encoding 5′-nucleotidases specific for purine nucleotides in industrially important *Bacillus* species, “shotgun” cloning and the direct selection of recombinant clones grown in purine nucleosides at inhibitory concentrations were performed in the *Escherichia coli* GS72 strain, which is sensitive to these compounds. As a result, orthologous *yitU* genes from *Bacillus subtilis* and *Bacillus amyloliquefaciens*, whose products belong to the ubiquitous haloacid dehalogenase superfamily (HADSF), were selected and found to have a high sequence similarity of 87%. *B. subtilis* YitU was produced in *E. coli* as an N-terminal hexahistidine-tagged protein, purified and biochemically characterized as a soluble 5′-nucleotidase with broad substrate specificity with respect to various deoxyribo- and ribonucleoside monophosphates: dAMP, GMP, dGMP, CMP, AMP, XMP, IMP and 5-aminoimidazole-4-carboxamide-1-β-d-ribofuranosyl 5′-monophosphate (AICAR-P). However, the preferred substrate for recombinant YitU was shown to be flavin mononucleotide (FMN). *B. subtilis* and *B. amyloliquefaciens yitU* overexpression increased riboflavin (RF) and 5-aminoimidazole-4-carboxamide ribonucleoside (AICAR) accumulation and can be applied to breed highly performing RF- and AICAR-producing strains.

**Electronic supplementary material:**

The online version of this article (10.1007/s00253-020-10428-y) contains supplementary material, which is available to authorized users.

## Introduction

*Bacillus subtilis*, *Bacillus amyloliquefaciens*, and other *Bacillus* species are gram-positive bacteria widely used for the production of enzymes, recombinant proteins, antimicrobial components (peptide and lipopeptide antibiotics and bacteriocins), insecticides, adsorbents, surfactants, and other industrially important biochemicals such as d-ribose, vitamins, purine nucleosides, and poly(gamma-glutamic acid) (Schallmey et al. 2004; Abriouel et al. 2011; Liu et al. 2013).

The main desirable features for the application of many *Bacillus* species as microbial cell factories are their generally recognized as safe (GRAS) status, probiotic properties, absence of exotoxins and endotoxin production, fully sequenced genomes, well-studied secretion pathways, and fairly simple cultivation conditions; their available transcriptome, metabolome, and proteome analysis data, and advanced genetic engineering tools are suitable for use with these species. *B. subtilis* and *B. amyloliquefaciens* strains have been successfully designed to produce riboflavin (RF), adenosine, inosine, guanosine, and 5-aminoimidazole-4-carboxamide ribonucleoside (AICAR), which are widely used in food technology and the pharmaceutical industry (Stepanov et al. 1984; Perkins et al. 1999; Asahara et al. 2010; Lobanov et al. 2011; Sheremet et al. 2011; Zhang et al. 2015). Since the compounds listed can be synthesized from their immediate phosphorylated precursors, flavin mononucleotide (FMN), AMP, IMP, GMP, and 5-aminoimidazole-4-carboxamide-1-β-d-ribofuranosyl 5′-monophosphate (AICAR-P), respectively, the construction of industrial producers requires not only enhanced metabolic flux towards the biosynthesis of these phosphorylated compounds but also the oversynthesis of enzymes with respective phosphatase or 5′-nucleotidase activity. 5’-Nucleotidases (EC 3.1.3.5) are enzymes that catalyze the hydrolytic dephosphorylation of 5′-ribonucleotides and 5′-deoxyribonucleotides to nucleosides and phosphate. These enzymes are widely distributed among all domains of life (Zimmermann 1992). Most well-studied soluble 5′-nucleotidases belong to the ubiquitous haloacid dehalogenase superfamily (HADSF) and have been shown to be involved in purine and pyrimidine salvage pathways, nucleic acid repair, cell-to-cell communication, signal transduction, etc. (Bianchi and Spychala 2003; Hunsucker et al. 2005; Borowiec et al. 2006). HADSF members, which are multifunctional enzymes with 5′-nucleotidase activity expressed by bacteria, control the intracellular concentrations of key phosphorylated metabolites and thereby participate in regulating cellular metabolism. The identification and investigation of these enzymes are important from both fundamental and applied points of view.

Despite the essential role of soluble 5′-nucleotidases in bacterial metabolism and the design of industrially important strains, little information about the functions of these enzymes from *Bacillus* species could be found in the literature. Terakawa and coauthors reported the 5′-nucleotidase activities of several *B. subtilis* proteins (YqeG, YcaA, YutF, YcsE, and YktC) (Terakawa et al. 2016) homologous to earlier described *E. coli* multifunctional enzymes that exhibit 5′-nucleotidase activity with respect to a remarkably broad and overlapping substrate spectrum (Matsuhisa et al. 1995; Kuznetsova et al. 2006). A HADSF member from *B. subtilis*, the 5′-nucleotidase YutF, was found to hydrolyze various purine and pyrimidine 5′-nucleotides, showing a preference for 5′-nucleoside monophosphates and, specifically, 5’-XMP (Zakataeva et al. 2016). Recently, enzymes with phosphatase and 5′-nucleotidase activities belonging to the HADSF were shown to catalyze essential steps in the biosynthesis of the key cellular metabolites serine and RF. Thus, YsaA from *B. subtilis* was found to be a phosphoserine phosphatase, the enzyme that catalyzes the final step of serine biosynthesis (Koo et al. 2017). Another HADSF member, *B. subtilis* YcsE, was shown to catalyze the dephosphorylation of 5-amino-6-ribitylamino-2,4(1H,3H)-pyrimidinedione 5′-phosphate (ARPP), forming the pyrimidine precursor of RF, 5-amino-6-ribitylamino-2,4(1H,3H)-pyrimidinedione (Sarge et al. 2015). Moreover, screening of 13 putative HADSF members from *B. subtilis* revealed that two additional proteins, YwtE and YitU, can catalyze the same reaction at appreciable rates (Sarge et al. 2015). Recently, a homologue of YwtE and YcsE, *B. subtilis* PhoC, which is probably involved in the phosphosugar stress response, was characterized (Morabbi Heravi et al. 2019).

In the present study, to search for genes encoding 5′-nucleotidases specific to purine nucleotides in *B. subtilis* and *B. amyloliquefaciens*, “shotgun” cloning and the direct selection of recombinant clones grown with purine nucleosides at inhibitory concentrations were performed in the *E. coli* GS72 strain, which is sensitive to these compounds. As a result, the *yitU* gene was selected, and its product was characterized as a 5′-nucleotidase with broad substrate specificity with respect to various deoxyribo- and ribonucleoside monophosphates. The preferred substrate for YitU was shown to be the redox-active coenzyme FMN. Furthermore, the application of *yitU* overexpression for the design of industrially important RF- and AICAR-producing strains was demonstrated.

## Materials and methods

### Bacterial strains and plasmids

The bacterial strains and plasmids used in this study are shown in Table 1. The primers used in this study are shown in Supplementary Table S1. *E. coli* was used as a host for cloning and protein expression. The *B. subtilis* and *B. amyloliquefaciens* strains, except for strain AJ1991purH::spc, were constructed using pNZT1-based delivery plasmids and a two-step replacement recombination procedure (Zakataeva et al. 2010), as described in Table 1. Single crossover was maintained by erythromycin (Em) resistance. Strain AJ1991purH::spc, in which the *spc* cassette was inserted into *purH*, was constructed by allele replacement (due to double crossover events) using the delivery plasmid pHY300PLK-purH::Sp.

**Table 1:** Bacteria and plasmids used in this study

Strain or plasmid	Relevant characteristics^a^	Source or description^b^
*Escherichia coli* strains		
GS72	TG1 Δ*deoD gsk-3*	Gronskiy et al. 2005
TG1	*supE hsdΔ5 thi* Δ(*lac-proAB*)*/*F′ *tra*Δ36 *proA*^*+*^*B*^*+*^*lacI*^*q*^*lacZ*ΔM15	VKPM B5837
BL21(DE3)	Host for pET vectors. λDE3, *ompT*	Novagen (Merck Millipore, Darmstadt, Germany)
*Bacillus subtilis* strains		
168	*trpC2*	VKPM B1727 (Kunst et al. 1997)
Bs∆DEG	Derivative of 168; contains ∆*deoD*::*kan* Δ*pbuE*::*cat* Δ*pupG::spc*	VKPM B13486
BsC^+^	Derivative of 168; contains wild-type *trpC*	pNZT1-trpCwt → 168
BsC^+^ΔU	Derivative of BsC^+^; contains a 780-bp in-frame deletion in *yitU* (ΔU)	pNZT1-∆yitUBs → BsC^+^
*B. subtilis* 168 *Δrib*	Derivative of 168; contains the *kan* gene inserted into the *ribD* region, RF auxotroph, Km^R^	VKPM B13485
Y25	*B. subtilis* riboflavin-producing strain; contains *ribO335 ribC1* azg^R^ ros^R^	VKPM B9850
Y25∆U	Derivative of Y25; contains a 780-bp in-frame deletion in *yitU*	pNZT1-∆yitUBs → Y25
BS168 ΔyutF	Derivative of 168; contains a 351-bp in-frame deletion of *yutF*	Zakataeva et al. 2016
BS168 ΔyutF ΔU	Derivative of BS168 ΔyutF; contains a 780-bp in-frame deletion in *yitU*	pNZT1-∆yitUBs → BS168 ΔyutF
*Bacillus amyloliquefaciens* strains		
AJ1991	*B. amyloliquefaciens* inosine- and guanosine-producing strain; contains Ade^−^, Ile^−^, azg^R^	VKPM B8994
IAM1523	*B. amyloliquefaciens* K, wild-type	Zakataeva et al. 2010
IAM∆DG	Derivative of IAM1523; contains *deoD::Km pupG::Cm*	Successive disruption of the *deoD* and *pupG* genes in the IAM1523 chromosome using the delivery plasmids pNZT1-∆deoD::Km and pNZT1-∆pupG::Cm, respectively
AJ1991purH::spc	AICAR-producing strain, derivative of AJ1991; contains *purH*::*spc*	pHY300PLK-purH::Sp → AJ1991
AJΔU	Derivative of AJ1991purH::spc; contains a 753-bp in-frame deletion of *yitU*	pNZT1-∆yitU_Ba_ → AJ1991purH::spc
Plasmids		
pMW118	Low copy number vector, *ori* of pSC101, Plac lacZ’ Ap^R^	Nippon Gene, Tokyo, Japan
pMWAL1	Low copy number bireplicon *E. coli*–*B. subtilis* shuttle vector; based on the theta-replicating *B. subtilis* plasmid pBS72 and pMW118 plasmid; Ap^R^ (*E. coli*), Cm^R^ (*B. subtilis*, *B. amyloliquefaciens*)	Smirnov and Kotliarova 2015
pMWAL1-yitU_Ba_	pMWAL1 derivative; contains a 1217-bp *Xba*I-*Sac*I fragment of the *B. amyloliquefaciens* IAM∆DG chromosome with the *yitU* gene and its upstream region (for *yitU*_Ba_ expression under control of its own regulatory elements)	The DNA fragment of IAM∆DG was PCR amplified (primer pair yitU+Xba/yitU-Sac), digested with *Xba*I-*Sac*I, and cloned into *Xba*I-*Sac*I-digested pMWAL1
pMWAL1-yitU_Bs_	pMWAL1 derivative; contains a 1191-bp *Xba*I-*Sac*I fragment of the *B. subtilis* 168 chromosome, with the *yitU* gene and its upstream region (for *yitU*_Bs_ expression under control of its own regulatory elements)	The DNA fragment of 168 was PCR amplified (primer pair (+)yitU Sac Bs/(−)yitU Xba Bs), digested with *Xba*I-*Sac*I, and cloned into *Xba*I-*Sac*I-digested pMWAL1
pMWAL1-PyitU_Ba_-yitU_Bs_	pMWAL1 derivative; contains a 344 bp fragment of the *yitU*_Ba_ upstream region fused to promoterless *yitU*_Bs_	The DNA fragment of 168 was PCR amplified (primer pair (−)yitU Xba Bs/(+)PBam yitUBs) and fused using OE-PCR with a PCR-amplified fragment of the *B. amyloliquefaciens* IAM∆DG chromosome (primer pair (+)yitU seq1 Bam/(−)yitUBs PBam). Obtained fragment was digested with *Xba*I-*Sac*I and cloned into *Xba*I-*Sac*I-digested pMWAL1
pET-15b(+)	*E. coli* expression vector, Ap^R^	Novagen (Merck Millipore, Darmstadt, Germany)
pET15-yitU_Bs_	pET-15b(+) derivative for the production of YitU_Bs_	Coding sequence of 168 *yitU* was PCR amplified (primer pair (+)yitU Nco Bs/(−)yitU BHI Bs, digested with *Nco*I*-Bam*HI, and cloned into *Nco*I*-Bam*HI-digested pET-15b(+)
pET15-H6-yitU_Bs_	pET-15b(+) derivative for the production of YitU_Bs_ with an N-terminal hexahistidine tag	Coding sequence of 168 *yitU* was PCR amplified (primer pair (+)yitU His Bs/(−)yitU BHI Bs), digested with *Nco*I*-Bam*HI, and cloned into *Nco*I*-Bam*HI-digested pET-15b(+)
pKS1	Thermosensitive integration vector, Em^R^, Km^R^	Shatalin and Neyfakh 2005
pNZT1	pKS1 derivative, thermosensitive integration vector, Em^R^	Zakataeva et al. 2010
pNZT1-trpCwt	pNZT1 derivative to introduce wild-type *trpC*^+^ in 168	The DNA fragment of 168 was amplified using OE-PCR (primers (+)trpC Hind Bs/(−)trpCw splc and (−)trpC Pst Bs/(+)trpCw splc), digested with *Hin*dIII-*Pst*I, and cloned into *Hin*dIII-*Pst*I-digested pNZT1
pNZT1-∆yitU_Bs_	pNZT1 derivative to introduce Δ*yitU*_Bs_	The DNA fragment of 168 was amplified using OE-PCR (primers: (+)yitU delR Bs/(−)yitU seq1 Bs and (−)yitU del L Bs/(+)yitU seq1 Bs), digested with *Pvu*II-*Eco*RV, and cloned into *Eco*RV-*Sma*I-digested pNZT1
pNZT1-∆yitU_Ba_	pNZT1 derivative to introduce Δ*yitU*_Ba_	The DNA fragment of *B. amyloliquefaciens* IAM∆DG was amplified using OE-PCR (primers: (+)yitU Sal Bam/(−)yitU delL Bam and (−)yitU Pst Bam/(+)yitU delR Bam), digested with *Pst*I-*Sal*I and cloned into *Pst*I-*Sal*I-digested pNZT1
pNZT1-∆pupG::Cm	pNZT1 derivative to introduce Δ*pupG::cat*	The DNA fragment of *B. amyloliquefaciens* IAM1523 was PCR amplified (primer pair punA-Xho/punA-Pst), digested with *Xho*I-*Pst*I, and cloned into *Xho*I-*Pst*I-digested pNZT1, yielding pNZT1-pupG. Then, the *cat* gene was cut from pUC7Cm (Blatny et al. 1997) with *Sal*I-*Sma*I and cloned into the *Sal*I-*Bsp*68I sites of pNZT1-pupG, giving pNZT1-∆pupG::Cm
pKS1-∆deoD::Km	pKS1 derivative to introduce ∆*deoD*::*kan*	DNA fragments of *B. amyloliquefaciens* IAM1523 containing the 5′ end and 3′ end of *deoD* coding region were PCR amplified with primer pairs deoD1-Xho/deoD1-Hind and deoD1-Sma/deoD1-Bcu, respectively. PCR fragments were digested with *Xho*I-*Hin*dIII and *Sma*I-*Bcu*I, respectively, and successively cloned into their respective sites in pKS1, yielding pKS1-∆deoD::Km
pDG1726	Plasmid containing the *spc* antibiotic cassette	Guérout-Fleury et al. 1995
pHY300PLK	*E. coli*–*B. subtilis* shuttle vector, Ap^R^ (*E. coli*), Tc^R^ (*B. subtilis, B. amyloliquefaciens*)	Ishiwa and Shibahara-Sone 1986
pHY300PLK-purH	pHY300PLK derivative with cloned *purH*	The DNA fragment of *B. amyloliquefaciens* IAM1523 was PCR amplified (primer pair P24/P25), digested with *Xho*I-*Eco*RI, and cloned into *Xho*I-*Eco*RI-digested pHY300PLK
pHY300PLK-purH::Sp	pHY300PLK derivative to introduce Δ*purH::spc*	DNA fragment containing the *spc* cassette was cut from pDG1726 using *Eco*RV-*Hinc*II and cloned into *Eco*47III-digested pHY300-purH

^a^*Ap*^*R*^, ampicillin resistance; *Em*^*R*^, erythromycin resistance; *Cm*^*R*^, chloramphenicol resistance; *Spc*^*R*^, spectinomycin resistance; *Tc*^*R*^, tetracycline resistance; *Km*^*R*^, kanamycin resistance; *azg*^*R*^, 8-azaguanine resistance; *ros*^*R*^, roseoflavin resistance

^b^This work unless otherwise specified; *VKPM*, the Russian National Collection of Industrial Microorganisms; pNZT1-∆yitU_Bs_ → BsC^+^ denotes a strain constructed from BsC^+^ using the pNZT1-∆yitUBs plasmid; *PCR*, polymerase chain reaction; *OE-PCR*, overlap extension PCR

### Growth conditions and preparation of crude cell extracts

*E. coli* and *B. subtilis* were grown in Luria-Bertani (LB) or M9 minimal medium (Miller 1972) supplemented with d-glucose (0.4% for *E. coli* or 2% for *Bacillus* unless otherwise specified). When required, thiamine HCl (5 μg/ml), RF (25 μg/ml), tryptophan (50 μg/ml), casamino acids (0.1% (w/v)), ampicillin (Ap, 100 μg/ml), erythromycin (Em, 200 μg/ml for *E. coli* or 10 μg/ml for *Bacillus*), kanamycin (Km, 10 μg/ml), tetracycline (Tc, 10 μg/ml), spectinomycin (Spc, 100 μg/ml), or chloramphenicol (Cm, 7 μg/ml) was added to the medium. Solid medium was obtained by adding 20 g/l agar to liquid medium. All reagents were purchased from Sigma-Aldrich (Steinheim, Germany) unless otherwise specified.

To select 5′-nucleotidase genes by the “shotgun” technique, recombinant plasmids containing DNA fragments from genomic libraries were transferred into *E. coli* strain GS72, and the resulting transformants were grown in glucose M9 minimal medium supplemented with inhibitory concentrations of guanosine (50 μg/ml) or inosine (1500 μg/ml).

In agar diffusion assays, drops of cellular suspensions of the *B. subtilis* 168 strain containing plasmids pMWAL1, pMWAL1-yitU_Bs_, pMWAL1-yitU_Ba_ or pMWAL1-PyitU_Ba_-yitU_Bs_ were placed onto M9 plates supplemented with glucose and tryptophan (without RF) on which a suspension of the RF auxotrophic strain *B. subtilis* 168 *Δrib* had previously been spread. After 16 h of cultivation at 37 °C, the diameters of the growth halos of *B. subtilis* 168 *Δrib* around the plaques of control strain harboring empty vector and *yitU-*overexpressing strains were assessed. All experiments were performed in triplicate.

Extracellular AICAR accumulation in AICAR-producing strains was evaluated by tube fermentation as previously described (Sheremet et al. 2011), but the initial glucose concentration in the fermentation media was 60 g/l. AICAR concentration in the culture broth was determined using high-performance liquid chromatography (HPLC) as described (Sheremet et al. 2011). Glucose concentrations were determined by an enzymatic method using an enzyme electrode (BIOSEN C-line; EKF Diagnostic, Germany). Bacterial growth was assayed by measuring the optical density of the culture broth (OD_600_) using a spectrophotometer (UV-1800, Shimadzu, Kyoto, Japan) at 600 nm.

Extracellular RF accumulation in RF-producing strains and BsC^+^-based strains was evaluated by flask fermentation. Cells were incubated on glucose medium plates for 18 h at 34 °C and then resuspended in 40 ml of fresh M9 medium supplemented with glucose (1% for RF-producing strains and 0.4% for BsC^+^-based strains) to an OD_600_ of 0.3 (for RF-producing strains) or 0.1 (for BsC^+^-based strains). Cm was added to plasmid-containing strains. Strains were incubated in 750-ml flasks at 34 °C in a rotary shaker for 72 h (for RF-producing strains) or 192 h (for BsC^+^-based strains). Every 24 h, samples were taken from each strain and analyzed for biomass accumulation (OD_600_) and RF and glucose concentrations.

RF concentrations in culture broth were determined using a UPLC Acquity system (Waters, USA) with a fluorescence detector. Samples (5 μl) of appropriately diluted cell-free supernatants were applied to a Nucleosil 100-5 C18 MPN column (4 × 125 mm, 5 μm; Macherey & Nagel). The following solvent system was used at a flow rate of 0.7 ml/min: 25% (vol/vol) acetonitrile–50 mM formic acid–50 mM ammonium formate (pH 4.3). Detection was carried out with a fluorescence detector (excitation, 325 nm; emission, 513 nm; Waters Associates, Inc., USA).

To analyze culture broth by liquid chromatography-tandem mass spectrometry (LC-MS/MS), cells were grown for 70 h in 20 ml of M9 medium supplemented with 0.2% glucose and Cm in a rotary shaker in 750-ml flasks. For LC-MS/MS analysis, cell-free supernatants of the culture broth were used.

To prepare crude cell extracts, cells grown with aeration to mid-log phase in LB or M9 medium (*E. coli*) and M9 medium (*Bacillus*) supplemented with thiamine HCl and Ap for *E. coli* or tryptophan, casamino acids and Cm (when required) for *Bacillus* were harvested by centrifugation, washed with 0.9% NaCl, resuspended in 0.7 ml of buffer (50 mM Tris-HCl, pH 7.5, 5 mM MgCl_2_, 10% glycerol, 1 mM AEBSF), and then lysed by sonication (3× 60 s), following which debris was removed by centrifugation at 13.200×*g* for 20 min at 4 °C. The protein concentration in the crude extract was 3 mg/ml.

### DNA manipulation and genetic methods

All recombinant DNA manipulation was conducted according to standard procedures (Sambrook and Russell 2001) and the recommendations of the enzyme manufacturer (Thermo Scientific, Lithuania, Vilnius). Plasmid and chromosomal DNA was isolated using the Qiagen Miniprep kit (Germany, Hilden) and Qiagen DNA purification kit (Germany, Hilden), respectively, according to the manufacturer’s instructions.

Transformation of *B. subtilis* competent cells, E40 bacteriophage transduction to transfer plasmids into *B. amyloliquefaciens* cells, PCR amplification, and DNA sequence analyses were performed as previously described (Zakataeva et al. 2010). Primers were purchased from Evrogen (Moscow, Russia). All constructs were verified by DNA sequencing.

### Heterologous expression of YitU and purification

The pET15-H6-yitU_Bs_ expression construct was transferred into *E. coli* BL21(DE3). The recombinant hexahistidine-tagged YitU_Bs_ (Ht-YitU_Bs_) protein was overexpressed in the obtained transformants as previously described (Zakataeva et al. 2016) and purified by immobilized metal affinity chromatography on a HisTrap HP column (GE Healthcare) according to the manufacturer’s instructions. Imidazole-eluted recombinant protein was transferred to buffer A (50 mM Tris-HCl buffer, pH 7.1, 5 mM MgCl_2_, 20% glycerol) by gel filtration on a Sephadex G-25 column (Pharmacia) and stored at − 70 °C until required. The protein concentration was assayed using a Bio-Rad protein assay kit (Bio-Rad) with bovine serum albumin as a standard. Sodium dodecyl sulfate-polyacrylamide gel electrophoresis (SDS-PAGE) was performed using 15% polyacrylamide gels and subsequent staining with Coomassie brilliant blue R250.

Gel filtration analysis was performed on a Superose 6 Increase 10/300 GL column (GE Healthcare Life Sciences) in PBS (10 mM phosphate buffer, 140 mM NaCl, pH 7.4) according to the manufacturer’s recommendation. The column was calibrated using a sample from a molecular mass standard kit (Gel Filtration Markers Kit for Protein Molecular Weights 29,000–700,000 Da, Sigma-Aldrich, St. Louis, USA*).*

### Enzymatic assay

General phosphodiesterase activity was measured spectrophotometrically at 25 °C in a reaction mixture (0.5 ml) containing 50 mM Tricine buffer (pH 8.5), 0.5–5 mM Me^2+^ (Mg^2+^ or Mn^2+^), 5 mM bis(*p*-nitrophenyl) phosphate (bis-*p*NPP) or 5 mM *p-*nitrophenyl phosphorylcholine (*p*NPPC) as a substrate and purified Ht-YitU_Bs_ (3 μg) diluted in a stabilization buffer (50 mM Tris-HCl buffer, pH 7.0, 5 mM MgCl_2_, 20% glycerol, 1 mg/ml BSA). The reaction was initiated by substrate addition, and *p*-nitrophenol (*p*NP) production was monitored at 410 nm (ε_410 nm_ = 15,460 M^−1^ cm^−1^). The specific phosphodiesterase activity towards 1 mM flavin adenine dinucleotide (FAD) was assessed using shrimp alkaline phosphatase as an auxiliary enzyme as previously described (Podzelinska et al. 2009).

General phosphatase activity towards the artificial substrate *p*NPP (*p*NPPase) was assayed spectrophotometrically at 25 °C. The standard reaction mixture (0.5 ml) contained 50 mM imidazole buffer, pH 7.0, 5 mM MgCl_2_, 10 mM *p*NPP, and purified Ht-YitU_Bs_ (3 μg) or crude cell extract (0.1 mg of total protein). The reaction was initiated by the addition of *p*NPP and monitored by continuously following the production of *p*NP at 410 nm. No activity was detected in the control reaction, which excluded the enzyme.

Specific phosphatase (5′-nucleotidase) activity towards physiological substrates was assayed by the rate of inorganic phosphate (Pi) release. A standard reaction mixture (0.5 ml) contained 50 mM imidazole buffer, pH 7.0, 5 mM MgCl_2_, 3 mM or 15 mM substrate for purified Ht-YitU_Bs_ (from 0.08 to 3 μg), and crude cell extract (0.1 mg of total protein), respectively. The assay was initiated by substrate addition and carried out at 30 °C for 10 min. The reaction rate was linear under these conditions. The amount of released inorganic phosphate (Pi) was assessed by a previously described colorimetric method (Chen et al. 1956). For acid-labile substrates (all di- and triphosphates, sugar phosphates, NADP, pyridoxal 5-phosphate, phosphonoacetic acid, phosphoenolpyruvate, PRPP), Pi was assessed by the method of Cariani (Cariani et al. 2004). Pi concentrations were estimated from a standard curve obtained with KH_2_PO_4_. To exclude the influence of nonenzymatic factors, the background phosphate level was monitored in parallel using a control reaction without enzyme. The activity was calculated by subtracting nonspecific substrate hydrolysis measured in the absence of protein, which was no more than 5% of the total activity. One unit of activity was defined as 1 μmol of Pi released per minute at 30 °C.

The pH dependence of the phosphatase activity towards *p*NPP (10 mM) or 5’-GMP (3 mM) was determined in the presence of 5 mM MgCl_2_ and purified Ht-YitU_Bs_. The assays were performed in the following buffer systems (50 mM): MES buffer between pH 5.5 and 6.5, imidazole buffer between pH 6.0 and 7.5, Tris-HCl buffer between pH 7.1 and 8.9, and CHES buffer between pH 9 and 9.5.

The metal dependence of the phosphatase activity of purified Ht-YitU_Bs_ towards *p*NPP (10 mM) or 5’-IMP (3 mM) was determined in 50 mM imidazole buffer, pH 7.0, using various divalent metal ions (Mg^2+^, Mn^2+^, Co^2+^, Ni^2+^).

To determine the Michaelis constant (*K*_m_) and maximal initial velocity (*V*_max_), kinetic analyses were performed using the appropriate activity assay with at least ten different concentrations of substrate in the range of 0 to 20 mM for nucleotides and 0 to 3 mM for FMN. The measured activities were analyzed using the Lineweaver–Burk plot or Hill plot (for AICAR-P) with the nonlinear curve-fitting program GraphPad Prism 8 software (GraphPad Software, Inc., San Diego, CA, USA). All *k*_cat_ values correspond to the turnover number per monomer. All kinetic parameters were obtained from at least three measurements.

### LC-MS/MS analysis

Detection of RF in samples was performed by LC-MS/MS using an Acquity system with a Xevo TQD mass detector (Waters) and a previously described method (Guo et al. 2006) with the following modifications. Chromatographic separation was achieved with an Acquity UPLC BEH C18 (1.7 μ, 2.1 × 100 mm) column. UPLC conditions were set as follows: column temperature 30 °C, *λ* = 222 nm, injection volume 5 μl, flow rate 0.3 ml/min, buffers: [A], 5%, and [B], 95% methanol in water. The gradient was as follows: [B] was increased from 5 to 70% over 10 min, then held for 2 min at 70%, decreased to 5% over 0.5 min and held for 2.5 min at 5%. The MS/MS conditions were as follows: electrospray ionization (ESI), positive ion mode, multiple reaction monitoring mode, capillary voltage 3.5 kV, desolvation temperature 600 °C, source gas flow 800 L/H, cone gas flow 3 L/H, source temperature 150 °C, cone voltage 32 V, and collision energy 25 V. The precursor-to-product ion transitions *m*/*z* 377 →*m*/*z* 243, *m*/*z* 377 → *m*/*z* 198, *m*/*z* 377 → *m*/*z* 172, *m*/*z* 377 → *m*/*z* 117, and *m*/*z* 377 → *m*/*z* 99 were used for quantification. Standards were prepared by dissolving RF in Milli-Q water. The calibration range for the mass spectrometer was from 45 to 4500 μg/l. The limit of detection was 10 μg/l.

### Statistical analysis

Statistical analyses were performed using GraphPad Prism version 8 (GraphPad Software, San Diego, CA, USA). One-way ANOVA and Tukey’s multiple-comparison test were used to determine significant differences among sample means. Tests were considered to be statistically significant if *P* values lower than 0.05 were obtained.

## Results

### Search for *B. subtilis* 5′-nucleotidases using the selection of clones resistant to purine nucleosides

To identify genes encoding 5′-nucleotidases in *Bacillus* species, a method to exploit the hydrolytic dephosphorylation activity of the gene products was applied. This method was based on “shotgun” cloning followed by the direct selection of DNA fragments containing 5′-nucleotidase genes identified by the resistance of recombinant *E. coli* cells to the purine nucleosides guanosine and inosine at inhibitory concentrations.

The uptake of extracellular nucleosides at even high concentrations is not toxic for wild-type *E. coli* cells (Petersen 1999). However, the phosphorylation of intracellular guanosine (inosine) catalyzed by guanosine-inosine kinase (EC 2.7.1.73) encoded by the *gsk* gene leads to the formation of GMP (IMP) (Fig. S1). GMP is further converted to IMP, AMP, and ADP, which, at high concentrations, inhibit the activity of 5-phosphoribosyl-1-pyrophosphate (PRPP) synthetase (Willemoës et al. 2002), resulting in PRPP deficiency and growth arrest (Petersen 1999). Proper functioning of PRPP synthetase is essential for life because PRPP is the biosynthetic precursor of the amino acids histidine and tryptophan, as well as purine, pyrimidine, and pyridine (NAD+, NADP+) nucleotides. Growth arrest is prevented in wild-type *E. coli* cells by the degradation of guanosine (inosine) to guanine (hypoxanthine) and ribose-1-phosphate (catalyzed by purine nucleoside phosphorylase encoded by *deoD*) and feedback inhibition of guanosine-inosine kinase activity by GMP (Fig. S1). However, the addition of guanosine (inosine) to the growth medium of *E. coli* cells incapable of purine nucleoside cleavage (Δ*deoD*) and expressing feedback-resistant guanosine kinase (due to a *gsk-3* mutation) caused an uncontrolled increase in intracellular GMP (IMP) and then AMP/ADP pools, followed by PRPP synthetase inhibition and growth arrest (Petersen 1999). Based on these data, we hypothesized that the dephosphorylation of excess nucleotides via 5′-nucleotidase gene overexpression in the *gsk-3* Δ*deoD* strain would remove PRPP synthetase inhibition and rescue sensitivity to the purine nucleosides guanosine and inosine.

Therefore, to find genes encoding enzymes with 5′-nucleotidase activity in *B. subtilis* and *B. amyloliquefaciens*, genomic libraries for the *B. subtilis* Bs∆DEG (168 ∆*deoD* Δ*pbuE* Δ*pupG*) and *B. amyloliquefaciens* IAM∆DG (IAM1523 *deoD::Km, pupG::Cm)* strains were first obtained. Both strains contain deletions of purine nucleoside phosphorylase genes (unlike *E. coli*, these bacteria have two purine nucleoside phosphorylase genes, *deoD* and *pupG*) to exclude the selection of these genes in this search. Then, genomic DNA was digested with *Eco*RI and ligated to the *Eco*RI-digested low copy number vector pMW118 to obtain recombinant plasmids for the expression of cloned genes controlled by their own regulatory elements. The resulting recombinant plasmids containing DNA fragments from the genomic libraries were transferred into *E. coli* strain GS72 (TG1 Δ*deoD gsk-3*), which is sensitive to purine nucleosides due to *deoD* and *gsk-3* mutations, to select clones resistant to guanosine (50 μg/ml) and inosine (1500 μg/ml) at inhibitory concentrations. More than 50 plasmids in which DNA fragments ranging in size from 1600 to 6000 bp had been inserted were selected. These insertions were identified by sequence analysis, followed by an NCBI database sequence similarity search (Altschul et al. 1990). Plasmids conferring the highest level of resistance to purine nucleosides that simultaneously contained open reading frames (ORFs) encoding putative phosphatases were selected for further investigation. Identification of genes responsible for the resistance phenotype revealed the *B. subtilis* and *B. amyloliquefaciens yutU* genes (*yitU*_Bs_ and *yitU*_Ba_, respectively), which encode putative phosphatases. These genes were recloned into the low copy number *E. coli*/*B. subtilis* shuttle vector, pMWAL1, under the control of their own regulatory elements, yielding the plasmids pMWAL1-yitU_Ba_ and pMWAL1-yitU_Bs_, respectively. Resistance to inosine and guanosine conferred upon GS72 cells by these plasmids was confirmed (Supplementary Table S2). Moreover, pMWAL1-yitU_Ba_ and pMWAL1-yitU_Bs_ were also found to increase resistance to the purine analog 2,6-diaminopurine (DAP) (Supplementary Table S2).

In silico analysis of the *5*′-untranslated regions (UTRs) of *yitU*_Bs_ and *yitU*_Ba_ did not reveal sequences that exactly matched consensus sequences from known SigA promoters. However, according to published data (Nicolas et al. 2012), *B. subtilis yitU* is transcribed from the SigA promoter as part of a tricistronic transcript that also includes the downstream ORFs BSU_11136 and *yizC*, both of which have unknown functions (Supplementary Fig. S2). Indeed, no putative *Rho*-independent transcription terminators immediately downstream of the *yitU* ORF were predicted using the ARNold: finding terminators web server (http://rna.igmors.u-psud.fr/toolbox/arnold/index.php). Based on low-level matching with the optimum consensus sequence of the identified *yitU* promoter, moderate expression of this gene, at least during exponential growth, was suggested. The UTRs of *yitU*_Bs_ and *yitU*_Ba_ demonstrated differences in their promoter and Shine–Dalgarno (SD) sequences, suggesting that these genes are expressed at different levels (Supplementary Fig. S3). Indeed, the pMWAL1-yitU_Bs_ and pMWAL1-yitU_Ba_ plasmids, in which *yitU*_Bs_ and *yitU*_Ba_, respectively, are expressed under the control of their own regulatory elements, conferred different levels of resistance to purine nucleosides and DAP to GS72 cells (Supplementary Table S2). Moreover, pMWAL1-PyitU_Ba_-yitU_Bs_, which contained a DNA fragment in which the coding region of *yitU*_Bs_ was placed under control of the *yitU*_Ba_ UTR, conferred a higher level of resistance than pMWAL1-yitU_Bs_.

When the *yitU* gene was identified in our previous study (Yusupova et al. 2014), the *yitU* product was annotated in the NCBI protein database (http://www.ncbi.nlm.nih.gov/protein) as a putative phosphatase and assigned to the Cof-type HAD-IIB subfamily of the HADSF and Cluster of Orthologous Groups of proteins (COG) no. 0561 (hydroxymethylpyrimidine pyrophosphatase and other HAD family phosphatases, ftp://ftp.ncbi.nih.gov/pub/COG/COG2014/static/byCOG/COG0561.html) due to the presence of specific domains and its similarity with *E. coli* Cof hydrolase. The *yitU*_Bs_ and *yitU*_Ba_ genes possess a high nucleotide sequence similarity of 75.2%. The translated YitU_Bs_ and YitU_Ba_ proteins have 78.9% identical and 87.0% similar amino acid residues (Supplementary Fig. S4), suggesting an identical function for the YitU_Bs_ and YitU_Ba_ proteins.

### Heterologous expression and purification of YitU_Bs_

To characterize the biochemical properties of YitU, two variants of *yitU*_Bs_ (to translate YitU_Bs_ in its native form and as an N-terminally hexahistidine-tagged protein) were cloned into the expression vector pET15b(+), yielding the expression plasmids pET15-yitU_Bs_ and pET15-H6-yitU_Bs_, respectively. After the introduction of these plasmids into *E. coli* strain BL21(DE3), both proteins were produced in a soluble form. The electrophoretic patterns of total extracted proteins by SDS-PAGE revealed protein bands with molecular masses of approximately 30 kDa, which was consistent with the predicted molecular masses of YitU_Bs_ and Ht-YitU_Bs_ (30.6 and 31.9 kDa, respectively). Moreover, these bands were not detected in the control strain, which contained empty pET15b(+) vector (Supplementary Fig. S5).

**Fig. 1:**
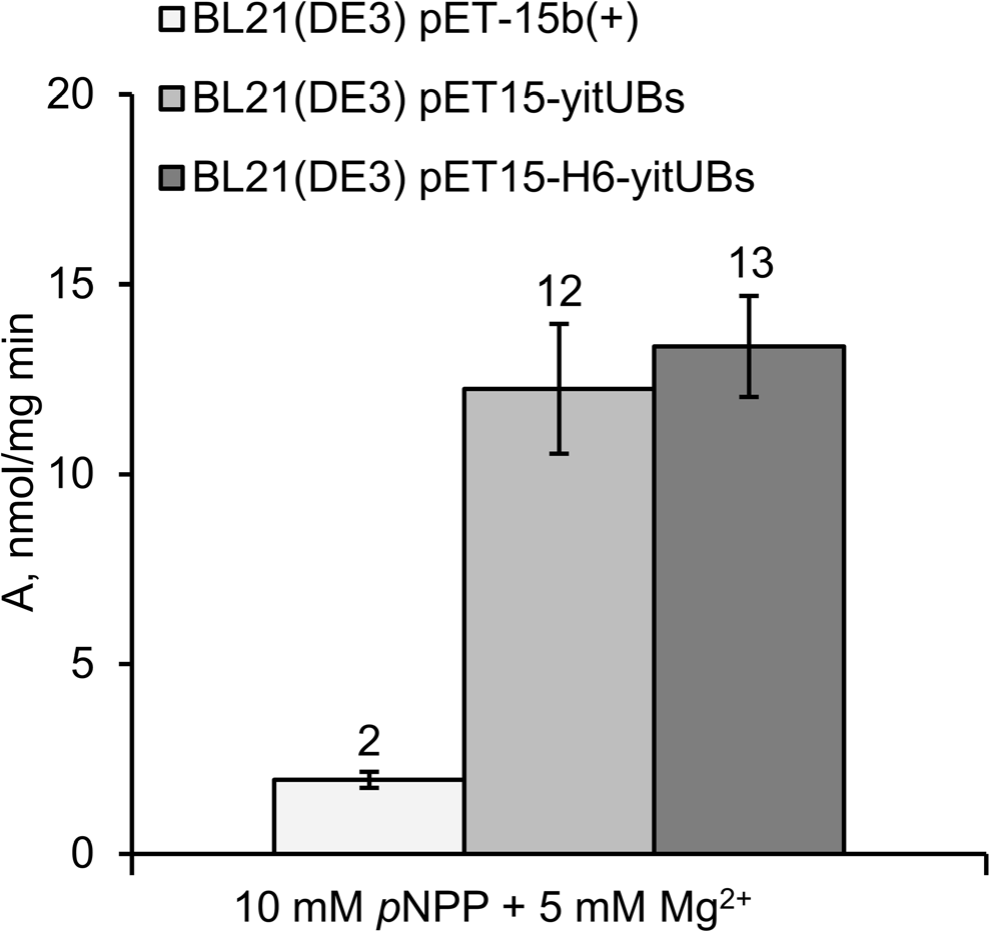
*p*NPPase activity in *E. coli* BL21(DE3) strains harboring empty vector (pET15b(+)) or plasmids with native *yitU*_Bs_ (pET15-yitU_Bs_) and N-terminally hexahistidine-tagged *yitU*_Bs_ (pET15-H6-yitU_Bs_). The results are expressed as the means of three independent experiments, and error bars indicate standard deviations

*p*NPPase activity towards the artificial substrate *p*NPP was assayed in BL21(DE3) (pET15b(+)), BL21(DE3) (pET15-yitU_Bs_) and BL21(DE3) (pET15-H6-yitU_Bs_) crude cell extracts (Fig. 1). YitU_Bs_ was shown to possess *p*NPPase activity. Moreover, the histidine tag at its N-terminus did not alter this activity. Therefore, further study was performed with the purified recombinant Ht-YitU_Bs_ protein.

The recombinant enzyme was purified to near homogeneity from the supernatant of disrupted BL21(DE3) (pET15-H6-yitU_Bs_) cells using immobilized metal affinity chromatography (Supplementary Fig. S5).

The Ht-YitU_Bs_ subunit structure was analyzed by gel filtration. The protein eluted as a single symmetric peak with a retention time that corresponded to a molecular mass of approximately 32 ± 5 kDa, suggesting that the active form of the enzyme is monomeric (Supplementary Fig. S6).

### Biochemical characterization of recombinant Ht-YitU_Bs_

General phosphatase screening with respect to artificial chromogenic substrates demonstrated that Ht-YitU_Bs_ has no activity towards bis-*p*NPP and *p*NPPC (contrary to *p*NPP), suggesting the absence of phosphodiesterase activity. The optimum pH for Ht-YitU_Bs_ was estimated to be 7.0 in 50 mM imidazole buffer with *p*NPP and GMP as artificial and physiological substrates, respectively (Supplementary Fig. S7). Similar to other members of the HADSF, Ht-YitU_Bs_ absolutely requires Mg^2+^ for its activity. The optimal concentration of Mg^2+^ was shown to be 5 mM (Supplementary Fig. S8). A maximum *p*NPPase activity of 160 nmol/mg min was observed in imidazole buffer, pH 7.0, in the presence of 5 mM MgCl_2_.

Under optimal conditions, the phosphatase activity of purified Ht-YitU_Bs_ with respect to a wide spectrum of physiological substrates (deoxyribo- and ribonucleoside tri-, di-, and monophosphates; sugar phosphates; and other phosphorylated metabolites) was evaluated as described in the “Materials and methods” section. Ht-YitU_Bs_ demonstrated the highest activity towards deoxyribo- and ribonucleoside monophosphates (Table 2). FMN, dAMP, GMP, dGMP, CMP, AMP, XMP, IMP, and AICAR-P proved to be its preferred substrates.

**Table 2:** Activity of purified Ht-YitU_Bs_ towards various substrates

Substrate	*A* (μmol/mg min^1^)	Source or reference
FMN (0.1 mM)	24.8 ± 3.6	
	17 ± 2	Sarge et al. 2015
ARPP (0.3 mM)	1.7 ± 0.4	
dAMP	13.1 ± 1.9	
GMP	12.1 ± 1.7	
CMP	10.9 ± 1.5	
AMP	9.7 ± 1.4	
dGMP	9.5 ± 1.6	
XMP	8.4 ± 1.4	
IMP	6.4 ± 0.9	
AICAR-P	2.8 ± 0.6	
2’AMP	2.2 ± 0.4	
CDP	1.4 ± 0.4	
UMP	1.3 ± 0.3	
GDP	1.3 ± 0.4	
6-Phospho-gluconate	1.2 ± 0.3	
IDP	0.90 ± 0.19	
Pyridoxal 5-phosphate	0.78 ± 0.12	
NADP	0.75 ± 0.11	
Ribose-5-phosphate	0.54 ± 0.08	
TDP	0.52 ± 0.07	
Mannose 6-phosphate	0.52 ± 0.07	
3’AMP	0.37 ± 0.06	
Glucose 6-phosphate	0.33 ± 0.05	
ADP	0.32 ± 0.05	
Fructose 6-phosphate	0.29 ± 0.04	
ITP	0.21 ± 0.04	
Erythrose 4-phosphate	0.19 ± 0.03	
CTP	0.16 ± 0.03	
GTP	0.14 ± 0.02	
Phosphoribosyl pyrophosphate	0.13 ± 0.02	
UDP	0.13 ± 0.02	
ATP	0.12 ± 0.02	
FAD (1 mM)	< 0.03	
UTP	< 0.03	
Phosphoenolpyruvate	< 0.03	
Glucose 1-phosphate	< 0.03	
Phosphonoacetic acid	< 0.03	

^1^The rates of substrate (3 mM unless otherwise specified) hydrolysis by purified Ht-YitU (0.12 μg) were measured as described in the “Materials and methods” section. The specific activity is presented as μmoles of Pi released per min per milligram of protein. The results are expressed as the means of three independent experiments ± standard error of the mean

The kinetic parameters with FMN, dAMP, GMP, dGMP, CMP, AMP, XMP, IMP, and AICAR-P were studied (Table 3). Ht-YitU_Bs_ was shown to have low substrate specificity (*K*_m_ values in the mM concentration range) and modest catalytic efficiencies with respect to all tested substrates except for FMN, for which the Michaelis constant was almost three orders of magnitude lower, and the catalytic efficiency was two orders of magnitude higher than those of the other tested substrates. The kinetic behavior of the enzyme in the hydrolysis of the tested substrates, except AICAR-P, followed Michaelis-Menten kinetics. For AICAR-P, the kinetic curve indicated allosterism with a Hill coefficient of 1.83 ± 0.15.

**Table 3:** Kinetic parameters of Ht-YitU for its preferred substrates

Substrate	*K*_m_^1^ (mM)	*V*_max_^1^ (U mg^−1^)	*k*_cat_ (s^−1^)	*k*_cat_/*K*_m_ (s^−1^ M^−1^)	Source or reference
FMN	0.096 ± 0.015	43.18 ± 1.52	22.97	2.39 × 10^5^	
ARPP	0.081 ± 0.006	–	0.88	1.09 × 10^4^	Sarge et al. 2015
dAMP	8.24 ± 0.42	49.35 ± 1.10	26.25	3.19 × 10^3^	
GMP	7.00 ± 0.29	39.97 ± 0.69	21.26	3.04 × 10^3^	
dGMP	5.61 ± 0.79	25.95 ± 1.33	13.80	2.46 × 10^3^	
CMP	17.45 ± 1.82	73.65 ± 3.94	39.18	2.25 × 10^3^	
AMP	14.32 ± 1.01	52.94 ± 2.13	28.16	1.97 × 10^3^	
XMP	21.74 ± 2.93	70.47 ± 6.56	37.49	1.72 × 10^3^	
IMP	17.81 ± 3.15	49.51 ± 4.53	26.34	1.48 × 10^3^	
AICAR-P	10.19 ± 1.50	21.20 ± 4.12	11.28	1.11 × 10^3^	

^1^The kinetic parameters were determined using the activity assay described in the “Materials and methods” section with at least ten different substrate concentrations. The results are expressed as the means of three independent experiments ± standard error of the mean

### Overexpression of *yitU* increased the extracellular accumulation of RF by wild-type *B. subtilis*

**Fig. 2:**
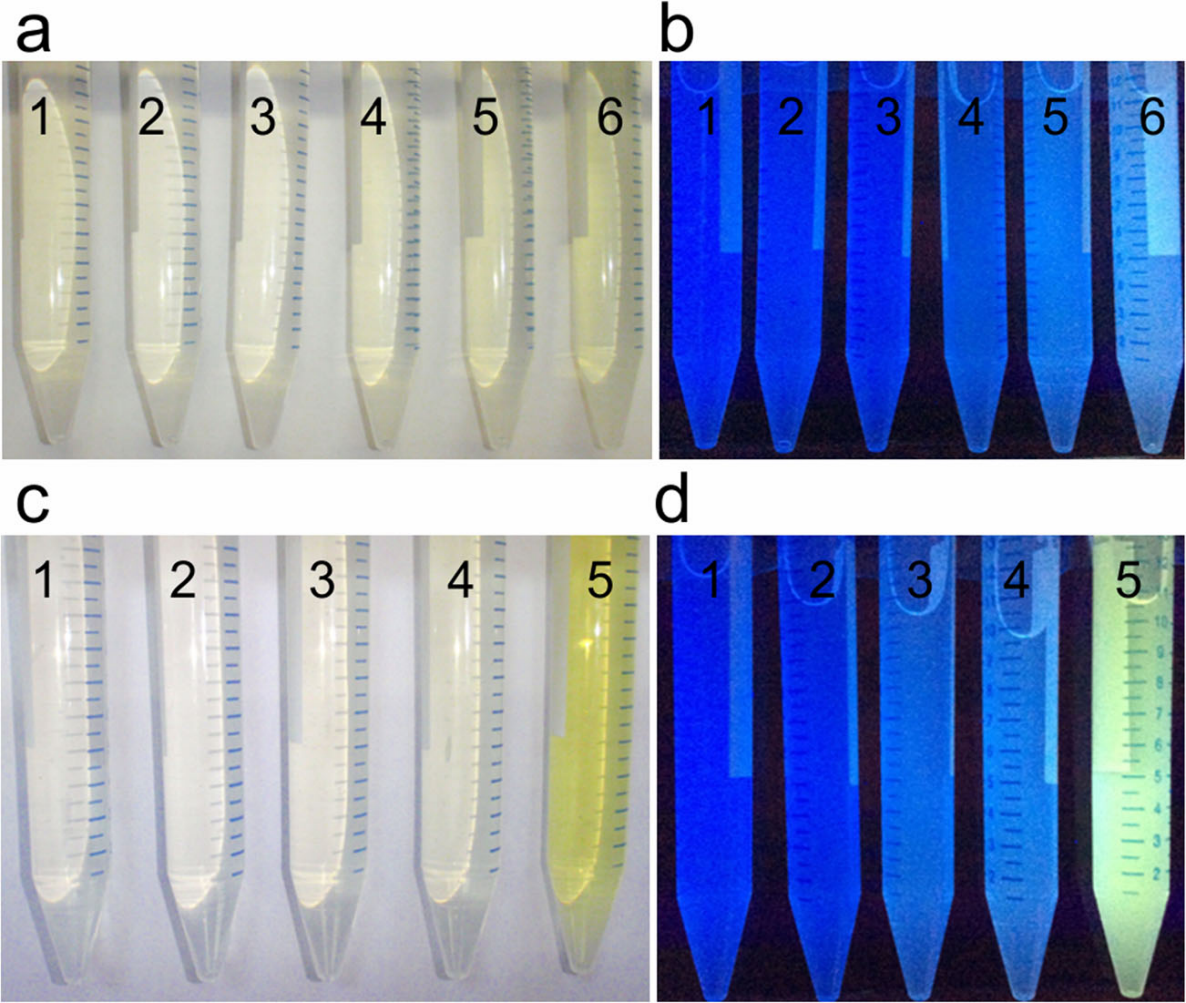
Effect of *yitU* overexpression on the accumulation of colored compounds in culture broth. **a**, **b** BsC^+^ derivatives: (1) BsC^+^, (2) BsC^+^∆U, (3) BsC^+^ (pMWAL1), (4) BsC^+^ (pMWAL1-yitU_Bs_), (5) BsC^+^ (pMWAL1-yitU_Ba_), (6) BsC^+^ (pMWAL1-PyitU_Ba_-yitU_Bs_). **c**, **d** BS168 ∆yutF derivatives: (1) BS168 ∆yutF, (2) BS168 ∆yutF (pMWAL1), (3) BS168 ∆yutF (pMWAL1-yitU_Bs_), (4) BS168 ∆yutF (pMWAL1-yitUBa), (5) BS168 ∆yutF (pMWAL1-PyitU_Ba_-yitU_Bs_). **b**, **d** The photo was captured under UV light

**Fig. 3:**
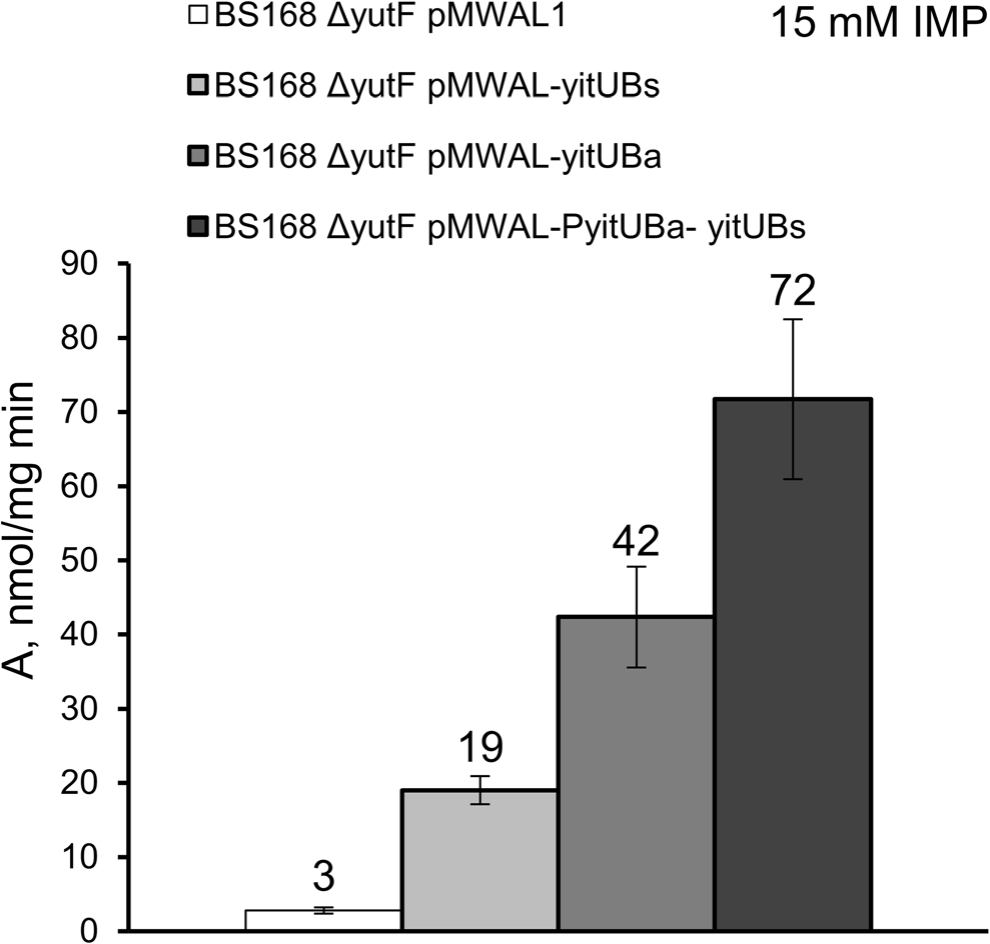
5’-Nucleotidase activity towards 15 mM IMP in *B. subtilis* strains overexpressing *yitU*_Bs_ and *yitU*_Ba._ The values are the means ± standard deviations of three independent experiments

Inactivation of *yitU* in the chromosome of the wild-type *B. subtilis* strain, BsC^+^ (*B. subtilis* 168 *trpC*^+^), had essentially no effect on cell growth and the glucose consumption rate during its cultivation in minimal medium (Supplementary Fig. S9). When the expression plasmids pMWAL1-yitU_Bs_, pMWAL1-yitU_Ba_, and pMWAL1-PyitU_Ba_-yitU_Bs_ or the empty vector pMWAL1 were transferred into BsC^+^, the resulting transformants were cultivated in minimal medium, and the culture broth of cells overexpressing *yitU* developed a yellow-green color (Fig. 2a). Moreover, the intensity of the color depended on the type of the *yitU* expression plasmid and was most intense in the case of the pMWAL1-PyitU_Ba_-yitU_Bs_ plasmid. Comparison of the 5′-nucleotidase activities in BS168 ∆yutF harboring pMWAL1, pMWAL1-yitU_Bs_, pMWAL1-yitU_Ba_, and pMWAL1-PyitU_Ba_-yitU_Bs_ showed that pMWAL1-PyitU_Ba_-yitU_Bs_ conferred the highest level of activity, suggesting the highest level of *yitU* expression due to the 5’ UTR of the *B. amyloliquefaciens* gene (Fig. 3). The BS168 ∆yutF strain, in which another 5′-nucleotidase gene, *yutF*, was disrupted, was used in this assay to exclude the impact of *yutF* on 5′-nucleotidase activity. Interestingly, we observed a more intense yellow-green color with BS168 ∆yutF (pMWAL1-PyitU_Ba_-yitU_Bs_) than with the BsC^+^ (pMWAL1-PyitU_Ba_-yitU_Bs_) strain (Fig. 2c vs Fig. 2a)*.*

**Fig. 4:**
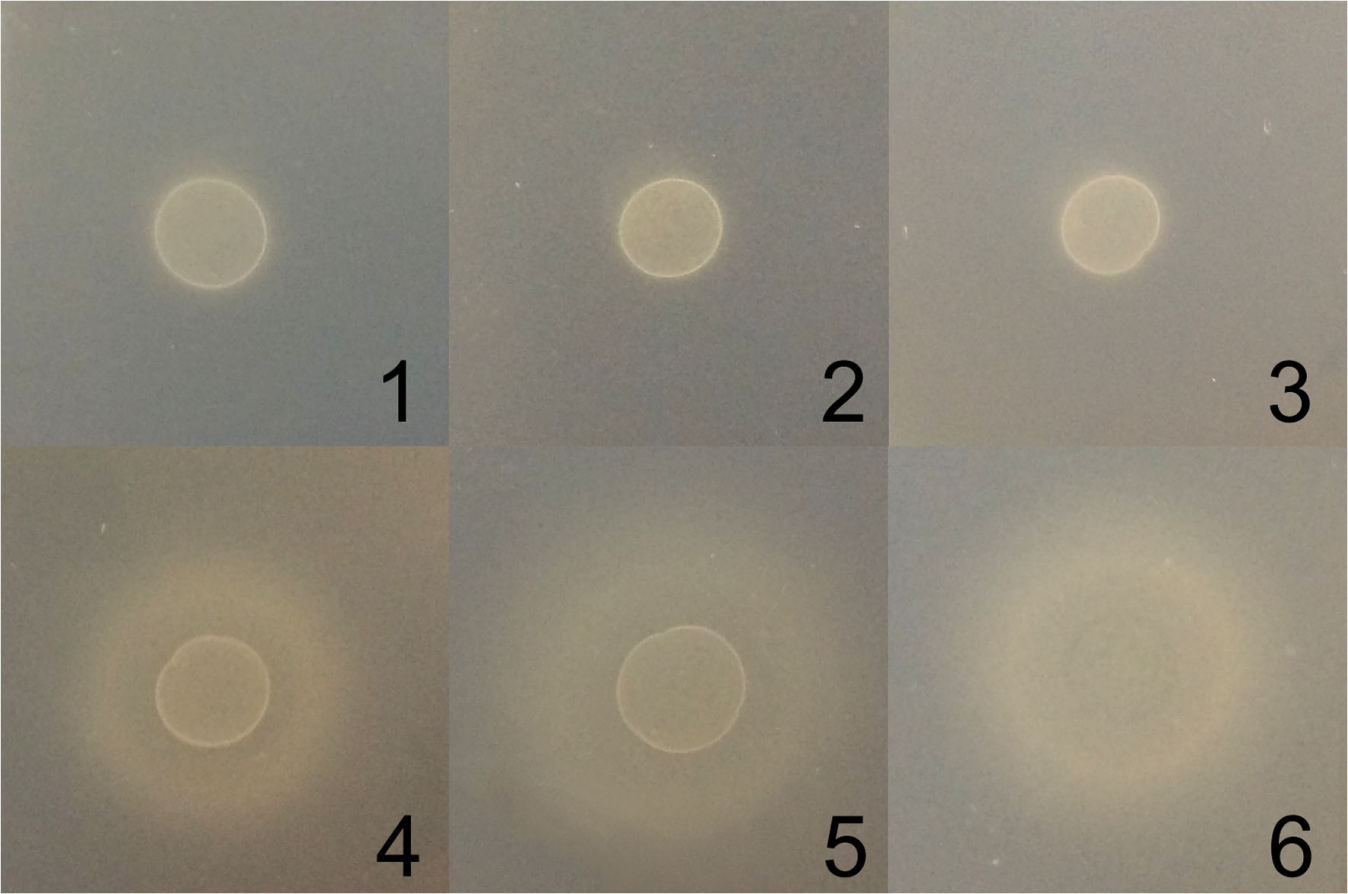
Agar diffusion assay. Halos of growth of the RF auxotrophic strain *B. subtilis* 168 *Δrib* around plaques of the following strains: (1) BS168 ∆yutF, (2) BS168 ΔyutF ΔU, (3) BS168 ∆yutF (pMWAL1), (4) BS168 ∆yutF (pMWAL1-yitU_Bs_), (5) BS168 ∆yutF (pMWAL1-yitU_Ba_), (6) BS168 (∆yutF pMWAL1-PyitU_Ba_-yitU_Bs_)

Since FMN is the preferred substrate of YitU, we supposed that the colored compound that accumulated in the culture broth is the product of FMN dephosphorylation, RF. Consistent with this suggestion, fluorescence of the colored culture broth was observed under UV light (Fig. 2b, d). Moreover, agar diffusion assays demonstrated that an RF auxotrophic strain, *B. subtilis* 168 *Δrib*, formed halos of growth around cells containing the pMWAL1-yitU_Bs_, pMWAL1-yitU_Ba_, and pMWAL1-PyitU_Ba_-yitU_Bs_ plasmids expressing *yitU*, most likely due to RF feeding (Fig. 4). Indeed, LC-MS/MS analysis of cell-free culture broth supernatants of BsC^+^ bearing the empty vector, pMWAL1, or the pMWAL1-PyitU_Ba_-yitU_Bs_ plasmid confirmed the presence of RF (Supplementary Fig. S10). Moreover, the RF concentration in the strain overexpressing *yitU* was 20 times higher than that in the strain harboring empty vector (2 mg/l vs 0.1 mg/l, respectively).

**Fig. 5:**
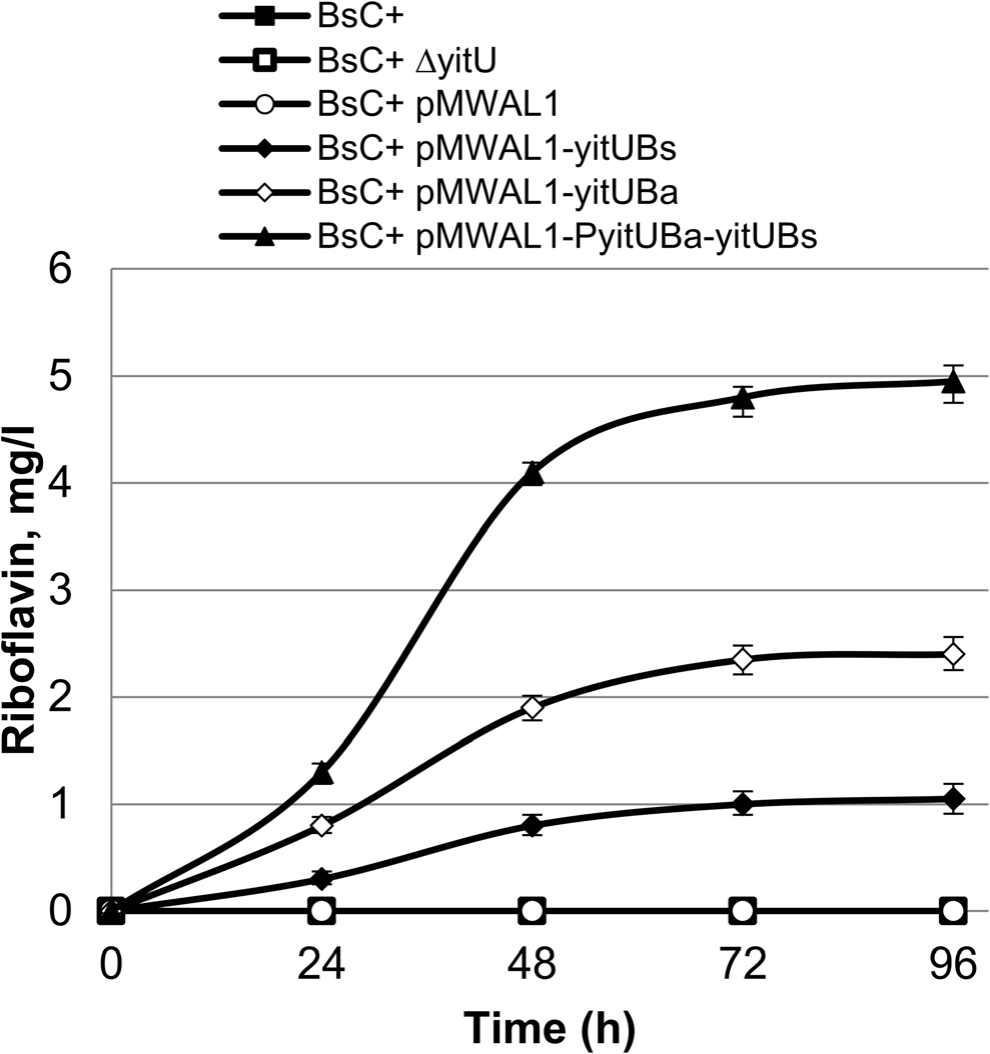
Extracellular RF accumulation in *B. subtilis* BsC^+^ harboring pMWAL1-yitU_Bs_, pMWAL1-yitU_Ba_, and pMWAL1-PyitU_Ba_-yitU_Bs_. The values are the means ± standard deviations of three independent experiments. Some error bars are smaller than the data point icons

The kinetics of RF accumulation in the culture broths of BsC^+^ cells in which *yitU* was disrupted or overexpressed were studied. In this experiment, the BsC^+^ strain and its ∆*yitU* derivative did not accumulate RF at detectable levels (Fig. 5, Supplementary Table S3). Derivatives of BsC^+^ harboring the pMWAL1-yitU_Bs_, pMWAL1-yitU_Ba_, and pMWAL1-PyitU_Ba_-yitU_Bs_ plasmids accumulated in culture broths from 1 to 5 mg/l RF. The plasmid expression of *yitU*_Bs_ under control of the *yitU*_Ba_ promoter region (pMWAL1-PyitU_Ba_-yitU_Bs_) led to a nearly fivefold increase in RF accumulation compared with the plasmid expression of *yitU*_Bs_ under native regulation (pMWAL1-yitU_Bs_).

### Disruption of *yitU* decreased, while enhancement of *yitU* expression increased, RF accumulation in an RF-producing strain

**Fig. 6:**
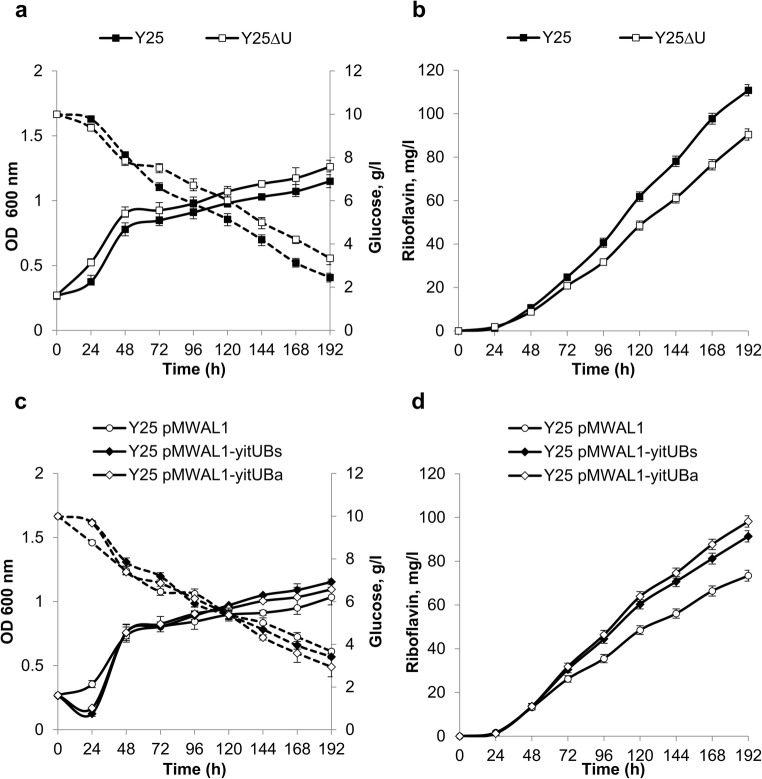
Influence of *yitU*_Bs_ deletion (**a**, **b**) and overexpression (**c**, **d**) on cell growth, glucose consumption (**a**, **c**), and extracellular accumulation of RF (**b**, **d**) in RF-producing *B. subtilis* strain Y25. Solid lines indicate growth (**a**, **c**) and RF accumulation (**b**, **d**), while dashed lines indicate glucose consumption (**a**, **c**). The values are the means ± standard deviations of three independent experiments. Some error bars are smaller than the data point icons

To further investigate the influence of *yitU* on RF production, the gene was disrupted and overexpressed in an RF-producing strain. *B. subtilis* Y25 can produce RF due to the increased expression of purine biosynthetic genes, overexpression of *rib* operon genes and deficiency of RF kinase activity (*ribC1*). This strain was obtained by the traditional selection of clones resistant to the purine analog 8-azaguanine and the RF analog roseoflavin (Mironov et al. 2002). Inactivation of *yitU* in strain Y25 reduced both RF accumulation and the glucose consumption rate at the productive phase but slightly increased the accumulated biomass (Fig. 6a, b). In contrast, *yitU* expression from plasmids pMWAL1-yitU_Bs_ and pMWAL1-yitU_Ba_ increased RF accumulation and slightly enhanced glucose consumption in strain Y25 at the productive phase (Fig. 6c, d).

### Disruption of *yitU* decreased, while enhancement of *yitU* expression increased, AICAR accumulation in an AICAR-producing strain

**Fig. 7:**
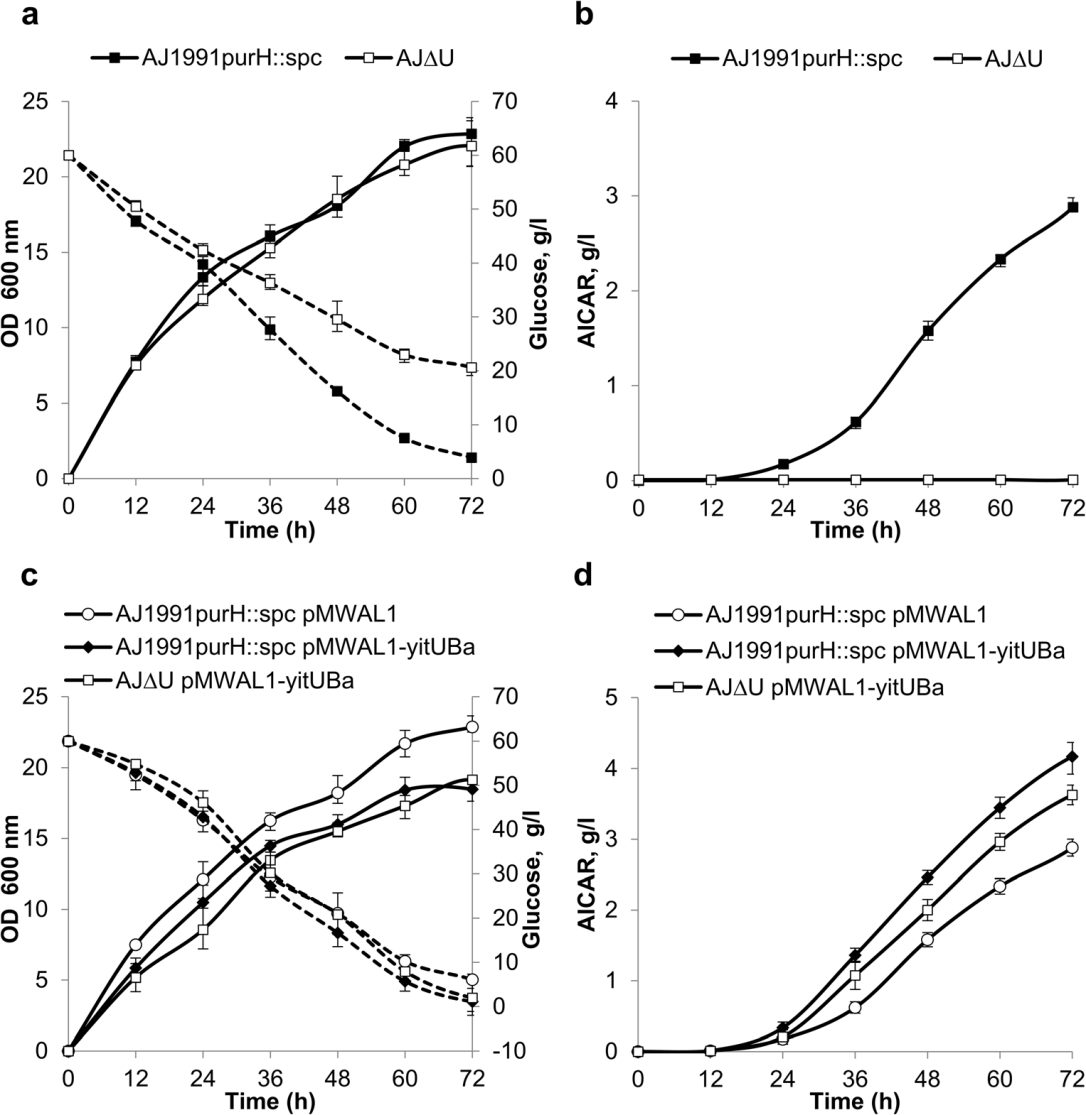
Influence of *yitU*_Ba_ deletion (**a**, **b**) and overexpression (**c**, **d**) on cell growth, glucose consumption (**a**, **c**), and extracellular accumulation of AICAR (**b**, **d**) in the *B. amyloliquefaciens* AICAR-producing strain AJ1991purH::spc. Solid lines indicate growth (**a**, **c**) and AICAR accumulation (**b**, **d**), while dashed lines indicate glucose consumption (**a**, **c**). The values are the means ± standard deviations of three independent experiments. Some error bars are smaller than the data point icons

Since we observed the specific behavior of Ht-YitU_Bs_ in AICAR-P hydrolysis, the effect of *yitU* disruption and overexpression on the performance of an AICAR-producing strain was studied. Strain AJ1991purH::spc can produce AICAR due to enhanced de novo purine biosynthesis and the blockade of the conversion of AICAR-P to IMP. Several derivatives of AJ1991purH::spc have been constructed. First, *yitU* was disrupted in the chromosome of AJ1991purH::spc, yielding strain AJΔU. The *yitU* overexpression plasmids pMWAL1-yitU_Bs_ and pMWAL1-yitU_Ba_ and the empty vector pMWAL1 (used as a control) were transferred into AJ1991purH::spc and AJΔU. The resulting strains were tested by test tube fermentation to evaluate the kinetics of cell growth, glucose consumption, and AICAR accumulation (Fig. 7). *yitU* deletion in AJ1991purH::spc had essentially no effect on cell growth but drastically decreased the glucose consumption rate and AICAR production (Fig. 7a, b).

*yitU*_Ba_ overexpression in AJΔU restored AICAR accumulation, which was lost in this strain due to *yitU* disruption (Fig. 7d). Moreover, compared with the control strain AJ1991purH::spc (pMWAL1), strain overexpressing *yitU*_Ba_ (AJ1991purH::spc (pMWAL1-*yitU*_Ba_)) demonstrated 1.6-fold increase in AICAR accumulation (Fig. 7d), less accumulated biomass, which nevertheless did not lead to a reduction in the glucose consumption rate (Fig. 7c) most likely due to more active product biosynthesis. The same effects on growth, glucose consumption, and AICAR accumulation were observed in AJ1991purH::spc and AJΔU due to *yitU*_Bs_ expression from pMWAL1-yitU_Bs_ (Supplementary Table S4, Supplementary Fig. S11).

## Discussion

Despite the important role of 5′-nucleotidases in cellular metabolism, only a few of these enzymes have been characterized in the gram-positive bacteria *B. subtilis* and *B. amyloliquefaciens*, the workhorses among industrial microorganisms. To identify genes encoding 5′-nucleotidases in *Bacillus* species, a search for genes homologous to earlier characterized 5′-nucleotidase genes in other bacteria, for example, *E. coli*, is often used as a suitable tool (Terakawa et al. 2016; Zakataeva et al. 2016). In this study, another method exploiting 5′-nucleotidase activity in gene products was applied. This method was based on “shotgun” cloning followed by the direct selection of DNA fragments containing 5′-nucleotidase genes due to the resistance of recombinant *E. coli* GS72 (TG1 *deoD gsk-3*) cells to the purine nucleosides guanosine and inosine at inhibitory concentrations.

Using this strategy, orthologous *yitU* genes were selected from genomic libraries of *B. subtilis* and *B. amyloliquefaciens* strains. Their products belong to the HADSF and have a high sequence similarity of 87%, suggesting the identical functions of these proteins. The *B. subtilis yitU* gene was produced in *E. coli* as an N-terminal hexahistidine-tagged protein, purified, and biochemically characterized as a soluble 5′-nucleotidase with a broad substrate specificity. Like many 5′-nucleotidases of the HADSF, YitU can dephosphorylate a wide range of substrates, including deoxyribo- and ribonucleotides. Among these compounds, the enzyme has the highest catalytic efficiency with respect to the monophosphates dAMP, GMP, dGMP, CMP, AMP, XMP, IMP, and AICAR-P. However, the preferred substrate with a Michaelis constant almost three orders of magnitude lower than the *K*_m_ values for the listed monophosphates was shown to be FMN (*K*_m_ = 0.096 mM). While this work was in progress, Sarge and coauthors reported that the products of the *B. subtilis* genes *ycsE*, *ywtE*, and *yitU* catalyze the dephosphorylation of ARPP (designated as 6 in the original publication) with high catalytic efficiency, forming the pyrimidine precursor of RF, 5-amino-6-ribitylamino-2,4(1H,3H)-pyrimidinedione (Sarge et al. 2015). The relatively high specific activities of YcsE, YwtE, and YitU towards FMN were also demonstrated (Sarge et al. 2015). In our experiments, we did not study the activity and kinetic characteristics of Ht-YitU_Bs_ towards ARPP due to the commercial inaccessibility of this compound and instead used data obtained by Sarge and coauthors for comparison. We found that although the Michaelis constants for ARPP and FMN were approximately the same, the catalytic constant (*k*_cat_) and catalytic efficiency (*k*_cat_/*K*_m_) of the enzyme for FMN were considerably higher than those for ARPP (Table 3). While Sarge and coauthors did not report the kinetic parameters for YitU with respect to FMN as a substrate, their data on the specific activity of purified YitU towards this compound are consistent with the value we report here (Table 2).

The expression of several enzymes (YcsE, YwtE, and YitU) with substrate specificity towards ARPP and FMN but different affinities for each of these substrates in *B. subtilis* might be necessary for fine-tuning cellular pools of the important flavins RF, FMN, and FAD. The main derivatives of RF, FMN and FAD, are redox-active coenzymes that associate with proteins to form flavoproteins. Flavoproteins function in a large variety of metabolic pathways, including electron transport, DNA repair, nucleotide biosynthesis, the synthesis of cofactors and heme groups, the β-oxidation of fatty acids, and amino acid catabolism (Abbas and Sibirny 2011). The role of flavoproteins in cellular redox metabolism is ensured by the ability of flavins to transfer electrons. Importantly, unlike other electron transfer cofactors, flavins can mediate both one-electron and two-electron transfer processes (Edwards 2014), making them one of the most important types of cofactors in cells. The intracellular concentrations, composition, and ratios of free flavins should be strongly regulated. FMN controls the biosynthesis and transport of RF by regulating related genes at the level of transcription or translation through a riboswitch mechanism (Gelfand et al. 1999; Winkler et al. 2002). YcsE, YwtE, and YitU, phosphatases with different flavin specificities, most likely exert their regulatory effects in conjunction with another enzyme involved in the conversion of RF to FMN and FMN to FAD, bifunctional flavokinase/flavin adenine dinucleotide synthetase (encoded in *B. subtilis* and *B. amyloliquefaciens* by *ribC*) (Mack et al. 1998).

ARPP dephosphorylation is commonly assumed not to be a bottleneck in RF production even in industrial producers that strongly overexpress other RF biosynthetic genes (Hümbelin et al. 1999; Perkins et al. 1999). In this study, we have shown that enhanced activity of the 5′-nucleotidase YitU in *B. subtilis* not only further elevated RF production in the RF-producing strain Y25 but also significantly increased RF accumulation in the wild-type strain BsC^+^, making it an RF producer. The positive effect of *yitU* overexpression on RF production can be attributed to at least two factors: enhanced de novo RF synthesis due to activation of one of its steps (ARPP dephosphorylation) and the enhanced conversion of FMN to RF, leading to a reduction in the FMN pool and thus upregulating RF biosynthesis.

Interestingly, the pMWAL1-PyitU_Ba_-yitU_Bs_ plasmid, which was shown to support the highest level of YitU activity, led to the severe retardation of Y25 (pMWAL1-PyitU_Ba_-yitU_Bs_) growth (data not shown), most likely due to a drastic deficiency in the redox-active cofactors FMN and FAD caused by the simultaneously impaired activity of bifunctional RF kinase/FMN adenylyltransferase (*ribC1*) and enhanced activity of FMN hydrolase. In contrast, the disruption of *yitU* in the chromosome of the Y25 strain reduced RF accumulation but increased cell growth, most likely due to a decrease in the conversion of FMN to RF, making FMN and FAD more available for various flavoproteins that catalyze important redox reactions in metabolism. In this study, we did not investigate the reason for increased RF accumulation due to *yitU* overexpression under the genetic background in which another 5′-nucleotidase gene, *yutF*, was deleted. The elimination of YutF function may have reduced the hydrolysis of some phosphorylated metabolites involved in RF biosynthesis.

AICAR-P, an intermediate in the purine nucleotide biosynthetic pathway and a byproduct of histidine biosynthesis, is a natural analog of AMP and a very important regulatory compound in bacteria, yeast, and humans. By both direct and indirect mechanisms, AICAR-P affects the biosynthesis of purines, thiamine, and histidine as well as one-carbon, carbohydrate, and lipid metabolism (Hürlimann et al. 2011; Daignan-Fornier and Pinson 2012; Bazurto et al. 2015; Ducker and Rabinowitz 2015; Malykh et al. 2018). In our study of the kinetic parameters of recombinant YitU, contrary to hydrolysis of the other tested substrates, which followed Michaelis-Menten kinetics, the kinetics of AICAR-P hydrolysis exhibited a sigmoidal behavior with a Hill coefficient of 1.83 ± 0.15, indicating positive cooperation. Gel filtration experiments showed that the active form of the enzyme is a monomer. Although cooperativity is traditionally observed in enzymes with multiple ligand-binding sites and/or multimeric assemblies, a few monomeric enzymes with single ligand-binding sites that display cooperativity have been described (Porter and Miller 2012). For example, among such enzymes is the best-studied mammalian glucokinase, which demonstrated a special type of allosteric regulation in which cooperativity was observed due to the rates of substrate transformation associated exclusively with conformational reorganization that occurs during substrate association (Storer and Cornish-Bowden 1976; Larion and Miller 2012).

The *K*_m_ value of YitU for AICAR-P as a substrate is in the millimolar concentration range and significantly higher than the physiological concentrations of AICAR-P (from 1.6 to 21.8 μM in exponentially grown yeast cells (Daignan-Fornier and Pinson 2012)). Therefore, YitU might hydrolyze AICAR-P under conditions in which this metabolite is oversynthesized. Moreover, positive cooperativity of the enzyme during AICAR-P hydrolysis could allow the cell to adapt to conditions in which the AICAR-P pool sharply increases. Indeed, in strain AJ1991purH::spc, in which the de novo purine biosynthetic pathway is enhanced and the conversion of AICAR-P to IMP is blocked, the plasmid expression of both *B. subtilis* and *B. amyloliquefaciens yitU* resulted in the increased accumulation of the product of AICAR-P hydrolysis, AICAR. The disruption of *yitU* in the chromosome of the AICAR producer AJ1991purH::spc had essentially no effect on cell growth but led to a decrease in AICAR production. This effect can be explained by the inhibition of purine biosynthesis by the drastically increased AICAR-P pool and supports the suggestion that YitU in *B. amyloliquefaciens* possesses major AICAR-P dephosphorylation activity.

To summarize, in this study, a new approach was used to search for 5′-nucleotidase genes, following which the *yitU* gene was selected. The product of this gene belongs to the HADSF and not only exhibits specificity for a wide spectrum of deoxyribo- and ribonucleoside monophosphates but also is involved in de novo (Sarge et al. 2015) and salvage RF biosynthesis (from FMN) pathways. Due to its ability to dephosphorylate the important redox-active cofactor FMN and an AMP analog with multiple regulatory functions, AICAR-P, YitU is involved in regulating cellular metabolism. It was also demonstrated for the first time that the overexpression of *yitU* can be successfully applied to breed highly performing RF- and AICAR-producing strains.

## Electronic supplementary material

ESM 1 (PDF 1.36 mb)
