# Supplementary material for: Expression and purification of the 5′-nucleotidase YitU from Bacillus species: its enzymatic properties and possible applications in biotechnology
Source: Appl Microbiol Biotechnol. 2020 Feb 10;104(7):2957–72. doi: 10.1007/s00253-020-10428-y (PMC7062661; doi:10.1007/s00253-020-10428-y)
Supplement: Supplementary file 1 — (PDF 1.36 mb) [file 253_2020_10428_MOESM1_ESM.pdf]

Applied Microbiology and Biotechnology

Supplementary Materials for

Expression and purification of the 5'-nucleotidase YitU from *Bacillus* species, its enzymatic properties and possible applications in biotechnology

Yuliya R. Yusupova[1], Victoria S. Skripnikova[1], Alexandr D. Kivero[1], Natalia P. Zakataeva[1*]

[1] Ajinomoto-Genetika Research Institute, 1-st Dorozhny Proezd, b.1-1, Moscow 117545, Russia.

*Corresponding author. Mailing address: Ajinomoto-Genetika Research Institute, 1-st Dorozhny Proezd, b.1-1, Moscow 117545, Russia E-mail: natalia_zakataeva@agri.ru, Phone: +74957803378 ext. (*) 415. Fax: +74953150640

Supplementary Materials

Table S1 Primers used in this study
Table S2 Effect of *yitU* overexpression on *E. coli* GS72 cell resistance to purine nucleosides, guanosine (GR), inosine (HxR) and purine analogue 2,6-diaminopurine (DAP)
Table S3 Extracellular riboflavin (RF) accumulation in *B. subtilis* BsC$^+$ and its derivatives after 96 h of incubation in minimal medium
Table S4 Extracellular 5-Aminoimidazole-4-carboxamide ribonucleoside (AICAR) accumulation in *B. amyloliquefaciens* AJ1991purH::spc and its derivatives after 72 h of incubation
Fig. S1 Schematic representation of the purine metabolism pathway in *E. coli*
Fig. S2 Schematic representation of *B. subtilis* 168 tricistronic transcript *yitU-BSU_11136-yizC*
Fig. S3 Sequence alignment of putative promoters of *yitU*$_{Bs}$ and *yitU*$_{Ba}$
Fig S4 Comparison of the deduced amino acid sequence of *B. amyloliquefaciens* YitU and *B. subtilis* YitU
Fig. S5 Expression of native YitU$_{Bs}$ and N-terminal hexahistidine-tagged Ht-YitU$_{Bs}$ in *E. coli*
Fig. S6 Gel-filtration chromatography of Ht-YitU$_{Bs}$
Fig. S7 pH-dependence of Ht-YitU$_{Bs}$ phosphatase activity towards GMP and *p*NPP in various buffers
Fig. S8 Activation of Ht-YitU$_{Bs}$ by divalent cations in various concentrations
Fig. S9 Effect of *yitU* deletion on cell growth and glucose consumption of *B. subtilis* BsC$^+$ (*B. subtilis* 168 *trpC*$^+$) during the cultivation in shaking flasks
Fig. S10 LC-MS/MS chromatograms at transitions 377→243, 377→198, 377→172, 377→117 and 377→99 for standard RF solution of 0.45 mg/l and 10 times diluted cell-free culture supernatants of BsC$^+$ (pMWAL1) and BsC$^+$ (pMWAL1-PyitU$_{Ba}$-yitU$_{Bs}$)
Fig. S11 Influence of *yitU*$_{Bs}$ overexpression on cell growth, glucose consumption and AICAR production in the *B. amyloliquefaciens* AICAR-producing strains AJ1991purH::spc and AJΔU

Table S1 Primers used in this study

Name	Sequence (5' to 3'), restriction sites are marked in bold	Application
(-)yitU Xba Bs	gtgggg**tctaga**aaggtttatgagtggg	pMWAL1-yitU$_{Bs}$
(+)yitU Sac Bs	cagc**gagctc**ttctccccgttccc	pMWAL1-yitU$_{Bs}$
yitU+Xba	tag**tctagat**gagtcgtctgttttagtatgg	pMWAL1-yitU$_{Ba}$
yitU-Sac	gc**gagctc**ttccgctgc	pMWAL1-yitU$_{Ba}$
(+)yitU Nco Bs	gagcttt**ccatgg**agacaaaaccc	pET15-yitU$_{Bs}$
(-)yitU BHI Bs	a**ggatcc**ttctttaaagtgagaaatattc	pET15-yitU$_{Bs}$<br>pET15-H6-yitU$_{Bs}$
(+)yitU His Bs	ta**ccatgg**gcagcagccatcatcatcatcatcacagcagcggcgagacaaaaccctatttaatcgc	pET15-H6-yitU$_{Bs}$
(+)trpC Hind Bs	ctggc**aagctt**gaatcggtttatc	pNZT1-trpCwt
(-)trpCw splc	gaagagaatcaataataaaatcttttctaagtacag	pNZT1-trpCwt
(-)trpC Pst Bs	gataa**ctgcag**ctcgcgccccatg	pNZT1-trpCwt
(+)trpCw splc	ctgtacttagaaaagattttattattgattctcttc	pNZT1-trpCwt
(+)yitU delR Bs	gagcttttcatggagacaaaacccgaatatttctcactttaaagaaggtccg	pNZT1-ΔyitU$_{Bs}$
(-)yitU seq1 Bs	ggcggaaaatctcaagcagg	pNZT1-ΔyitU$_{Bs}$
(-)yitU delL Bs	cggaccttctttaaagtgagaaatattcgggttttgtctccatgaaaagctc	pNZT1-ΔyitU$_{Bs}$

(+)yitU seq1 Bs	catatgcagtcaaagcgccaag	pNZT1-ΔyitU$_{Bs}$
(-)yitU Xba Bs	gtgggg**tctaga**aaggtttatgagtggg	pMWAL1-PyitU$_{Ba}$-yitU$_{Bs}$
(+)PBam yitUBs	acagcaaatcataaaggagttatttatggagacaaaaccctatttaatcg	pMWAL1-PyitU$_{Bam}$-yitU$_{Bs}$
(+)yitU seq1 Bam	gatcggaaaaaggctgaaaacg	pMWAL1-PyitU$_{Ba}$-yitU$_{Bs}$
(-)yitUBs PBam	cgattaaatagggttttgtctccataaataactcctttatgatttgctgt	pMWAL1-PyitU$_{Ba}$-yitU$_{Bs}$
(+)yitU Sal Bam	ctgac**gtcgac**tttgacccaatc	pNZT1-ΔyitU$_{Ba}$
(-)yitU del L Bam	ggagaaataggatgttaaaaatgcgaaggcgatgagataaggttttgtc	pNZT1-ΔyitU$_{Ba}$
(-)yitU Pst Bam	tgcat**ctgcag**aattggcgtacgc	pNZT1-ΔyitU$_{Ba}$
(+)yitU delR Bam	gacaaaaccttatctcatcgccttcgcatttttaacatcctatttctcc	pNZT1-ΔyitU$_{Ba}$
P24	cgcctcgccgaatttctcatc	pHY300PLK-purH
P25	aagggaattctttcggcggtgcggctc	pHY300PLK-purH
punA-Xho	cat**ctcgag**cggaagcgatcgcaattc	pNZT1-ΔpupG::Cm
punA-Pst	cat**ctgcag**tgcatctcgcacggattg	pNZT1-ΔpupG::Cm
deoD1-Xho	atactcgagagagctggccgctgacaat	pKS1-ΔdeoD::Km
deoD1-Hind	agct**aagctt**cccgtaaatccgtacattcc	pKS1-ΔdeoD::Km
deoD1-Sma	ta**cccggg**ttggcgctgcatactgtatc	pKS1-ΔdeoD::Km

deoD1-Bcu	tg**actagt**gcaagccccgtctgatgtt	pKS1-ΔdeoD::Km

Table S2 Effect of *yitU* overexpression on *E. coli* GS72 cell resistance to purine nucleosides, guanosine (GR), inosine (HxR) and purine analogue 2,6-diaminopurine (DAP)

**Strain**	**Minimal inhibitory concentration (MIC, µg/ml)***		
	GR	HxR	DAP
GS72 (pMWAL1)	<5	<500	<200
GS72 (pMWAL1-yitU$_{Bs}$)	15	500	300
GS72 (pMWAL1-yitU$_{Ba}$)	20	2000	400
GS72 (pMWAL1-PyitU$_{Ba}$-yitU$_{Bs}$	20	2000	400

*The MIC was determined as lowest concentration of an inhibitor preventing growth in minimal agar plates after 40 h incubation. The experiments were repeated three to five times and revealed consistent MICs

Table S3 Extracellular riboflavin (RF) accumulation in *B. subtilis* BsC$^+$ and its derivatives after 96 h of incubation in minimal medium

**Strain**	**OD$_{600}$**	**RF, mg/l**
BsC$^+$	0.4	≤0.1
BsC$^+$ΔU	0.5	≤0.1
BsC$^+$ (pMWAL1)	1.2	≤0.1
BsC$^+$ (pMWAL1-yitU$_{Bs}$)	1.2	1.1 ± 0.1
BsC$^+$ (pMWAL1-yitU$_{Ba}$)	0.9	2.4 ± 0.1
BsC$^+$ (pMWAL1-PyitU$_{Ba}$-yitU$_{Bs}$)	0.8	5.0 ± 0.1

Table S4 Extracellular 5-Aminoimidazole-4-carboxamide ribonucleoside (AICAR) accumulation in *B. amyloliquefaciens* AJ1991purH::spc and its derivatives after 72 h of incubation

**Strain**	**OD_{600}**	**AICAR, g/l**
AJ1991purH::spc	22.85	2.88 ±0.064
AJΔU	22.04	<0.01
AJ1991purH::spc (pMWAL1)	22.87	2.74 ± 0.08
AJ1991purH::spc (pMWAL1-$yitU_{Bs}$)	18.33	4.1 ± 0.03
AJ1991purH::spc (pMWAL1-$yitU_{Ba}$)	18.60	4.24 ± 0.23
AJΔU (pMWAL1-$yitU_{Bs}$)	19.65	3.72 ± 0.07
AJΔU (pMWAL1-$yitU_{Ba}$)	18.63	3.53 ± 0.20

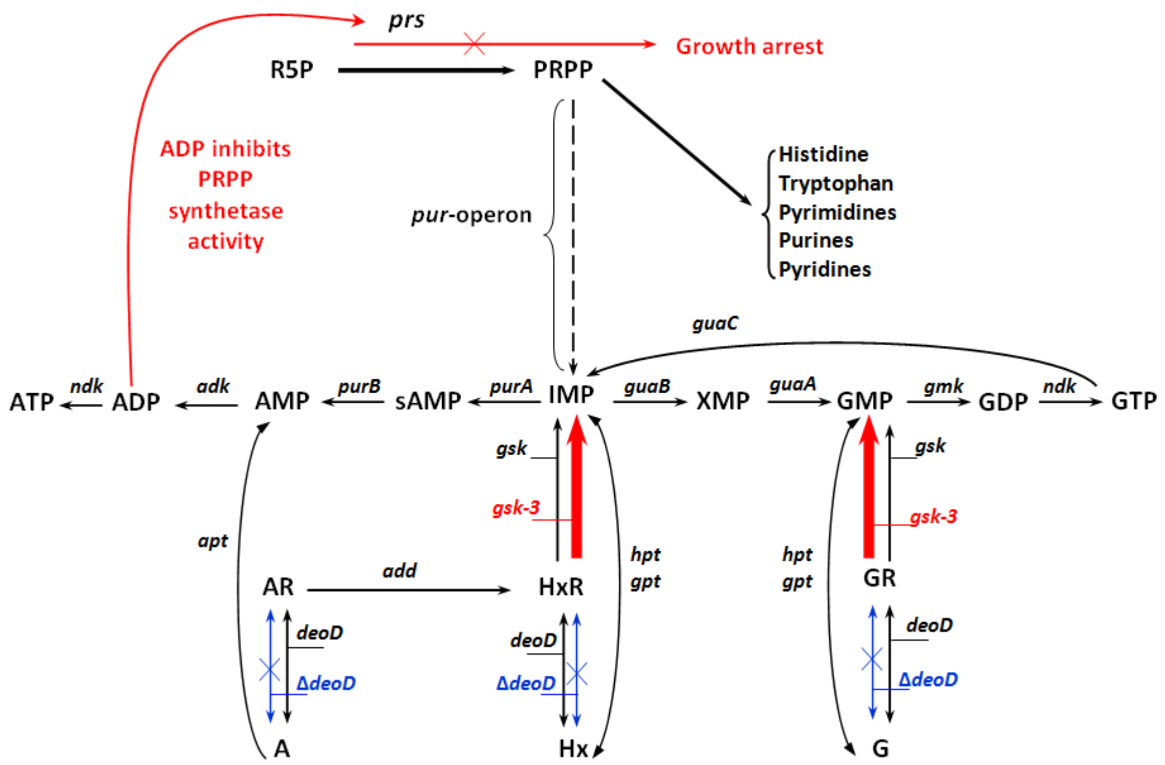

Fig. S1 Schematic representation of the purine metabolism pathway in *E. coli*. R5P – ribose-5-phosphate; PRPP – phosphoribosyl pyrophosphate; sAMP – succinyladenosine monophosphate; AR – adenosine; A – adenine; HxR – inosine; Hx – hypoxanthine; GR – guanosine; G – guanine. Genes and their corresponding encoded enzymes: *prs* – ribose-phosphate diphosphokinase; *gsk* – inosine/guanosine kinase; *deoD* – purine nucleoside phosphorylase; *ndk* – nucleotide diphosphate kinase; *adk* – adenylate kinase; *purB* – adenylosuccinate lyase; *purA* – adenylosuccinate lyase; *apt* – adenine phosphoribosyltransferase; *add* – adenosine deaminase; *guaB* – inosine 5'-monophosphate dehydrogenase; *guaA* – GMP synthetase; *hpt* – hypoxanthine phosphoribosyltransferase; *gpt* – xanthine-guanine phosphoribosyltransferase; *gmk* – guanylate kinase; *guaC* – GMP reductase. Red bold lines indicate enhanced GR (HxR) to GMP (IMP) conversion due to *gsk-3* mutation. Blue crossed arrows indicate blocked conversion due to *deoD* gene deficiency. Red crossed arrow indicates lack of PRPP synthetase activity resulted in PRPP deficiency and growth arrest. A red arrow indicates PRPP synthetase inhibition by ADP

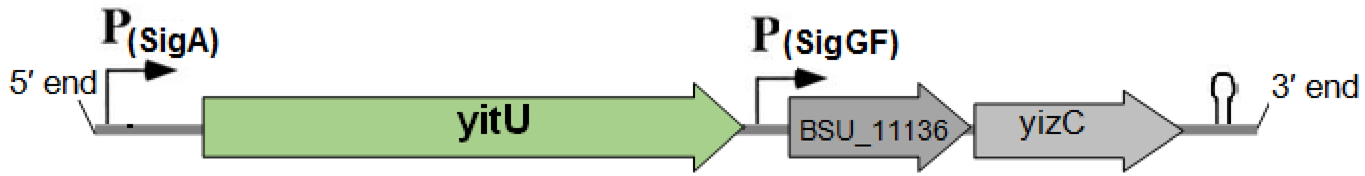

Fig. S2 Schematic representation of *B. subtilis* 168 tricistronic transcript *yitU-BSU_11136-yizC* based on published data (Nicolas et al. 2012; Ravikumar et al. 2018; http://www.subtiwiki.uni-goettingen.de/v3/gene/view/3E19806285A95F298C87732893383ABEF2695AF9)

```
                                                                                                                   SD         Met
tgtgcccctccttaggataaccataatcataagtgtaacaTGTAGACAataaagatggtaaAAAGCTACACTGTAAGTAAGgcaaaacaagcaaaatctaaggttaaaggagctttcatggag Bs
tgactccctccttaggataaccataatcataagtgtaacaTGTAGACAataaagatggtaaAAAGCTACACTGTACATAAGagaaaaacagcaaatcata------aaggagttatttatggag Ba
tgtgcccctccttaggataaccataatcataagtgtaacaTGTAGACAataaagatggtaaAAAGCTACACTGTAAGTAAGgcaaaacaagcaaaatctaa          putative promoter
```

Fig. S3 Sequence alignment of putative promoters of *yitU*$_{Bs}$ and *yitU*$_{Ba}$. Identical nucleotides for *B. subtilis* (Bs) and *B. amyloliquefaciens* (Ba) sequences are marked in yellow, non-identical nucleotides are marked in blue; putative SD region is shown by open box; putative –35 and –10 regions are marked in capital letters. Putative promoter sequence is shown based on published data (Nicolas et al. 2012)

```
YitU_Ba  METKPYLIALDLDGTLLKDDKTISPETARTIQLLTEAGHHICISTGRPFRSSSMYYDELG  60
YitU_Bs  METKPYLIALDLDGTLLKDDKTISENTLHTIQRLKDDGHYVCISTGRPYRSSSMYYQQME  60
         ************************ :* :*** *.: **::*******:*******:::

YitU_Ba  LTTPIVNFNGAFVHHPKDESWGRYHTSLSVDVVRQLAELTETYQIHNVLAEVIDDVYFHY  120
YitU_Bs  LTTPIVNFNGAFVHHPQDDSWGRYHTSLPLDVVKQLVDISESYNVHNVLAEVIDDVYFHY  120
         ****************:*:********* :***:**.:::*:*::***************

YitU_Ba  HDEHLIEAFHMNMANVTFGDLRETVKEDVTSVLIHAKEEDVADIRAHLSEVHAEVIDHRR  180
YitU_Bs  HDEHLIDAFNMNTTNVTVGDLRENLGEDVTSVLIHAKEEDVPAIRSYLSDVHAEVIDHRR  180
         ******:**:** :***.*****.: ***************  **::**:**********

YitU_Ba  WAAPWHVIEIIKHGMNKAVGLKKISDYYQVPRERIIAFGDEDNDLEMLRFAGCGVAMENG  240
YitU_Bs  WAAPWHVIEIIKSGMNKAVGLQKISDYYGVPRERIIAFGDEDNDLEMLEFAGCGVAMGNG  240
         ************ ********:****** *******************.******** **

YitU_Ba  TDEVKQAADRVTGSNEADGIAAFLTSYFSL                                270
YitU_Bs  IDAVKQIANRTTATNEEDGVARFLKEYFSL                                270
          * *** *:*.*.:** **:* **..****
```

Fig S4 Comparison of the deduced amino acid sequence of *B. amyloliquefaciens* YitU and *B. subtilis* YitU. Similar ('.' and ':') and identical ('*') amino acids are indicated. The following protein sequences were used (GenBank accession numbers are indicated in parentheses): YitU_Bs, putative phosphatase from *B. subtilis* (NP_388995.1); YitU_Ba putative phosphatase from *B. amyloliquefaciens* (AEB23217.1). The multiple sequence alignment was generated using CLUSTAL Omega. https://www.ebi.ac.uk/Tools/msa/clustalo/

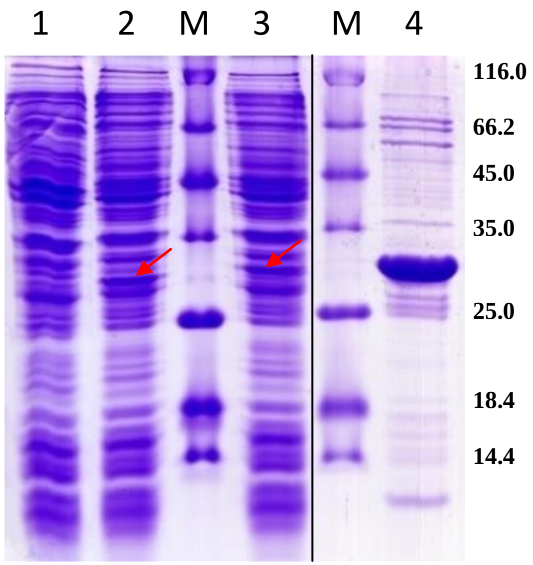

Fig. S5 Expression of native $YitU_{Bs}$ and N-terminal hexahistidine-tagged Ht-$YitU_{Bs}$ in *E. coli*. Lanes 1, 2 and 3 crude cellular lysates of BL21(DE3) harboring following plasmids: 1 – pET15b(+); 2 – pET15-$yitU_{Bs}$; 3 – pET15-H6-$yitU_{Bs}$. 27 µg of total protein. Lane 4: the purified Ht-$YitU_{Bs}$ product (7 µg). M – molecular mass standard (Unstained Protein Molecular Weight Marker, Thermo Scientific, Lithuania, Vilnius). Protein samples were separated by SDS-PAGE and stained with Coomassie Brilliant Blue

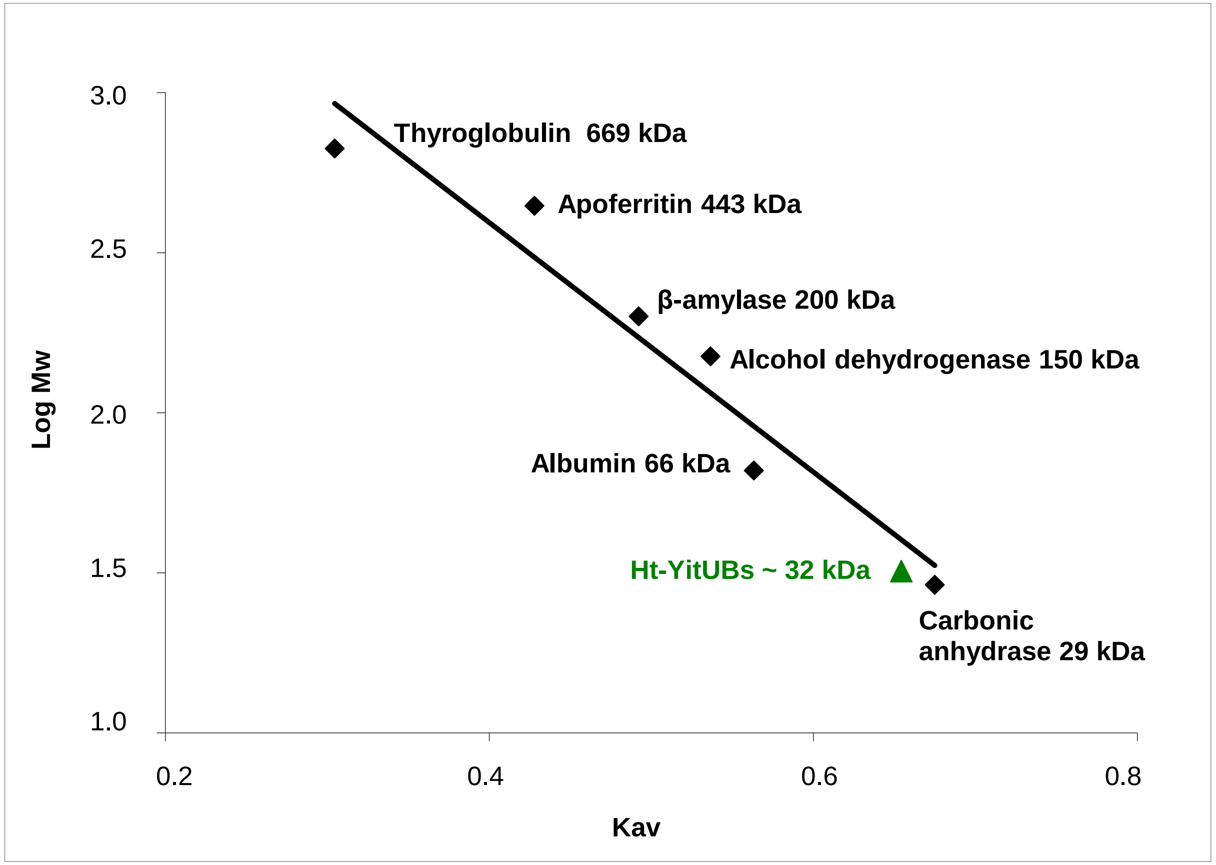

Fig. S6 Gel-filtration chromatography of Ht-YitU$_{Bs}$ (triangle) and reference proteins (diamond). Gel Filtration Markers Kit for Protein Molecular Weights 29,000-700,000 Da, Sigma-Aldrich, St. Louis, USA

a

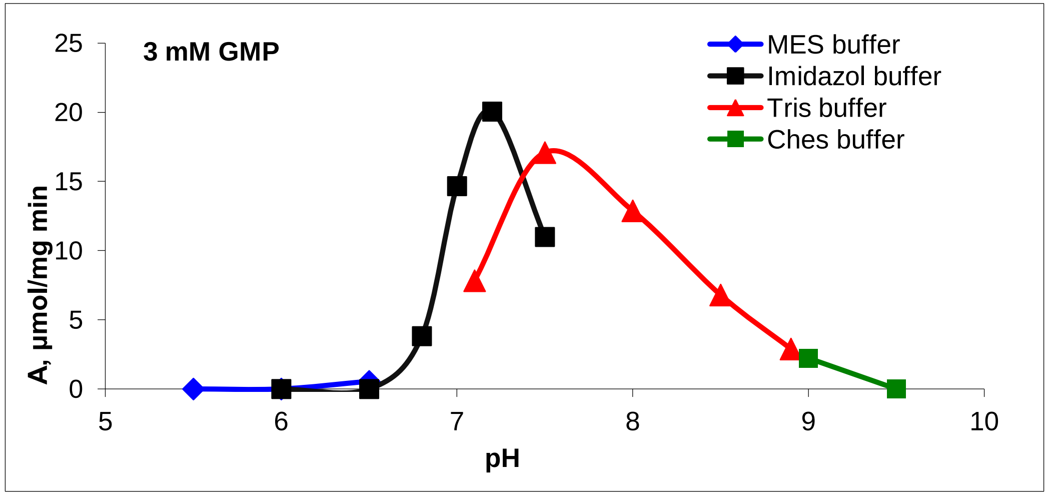

b

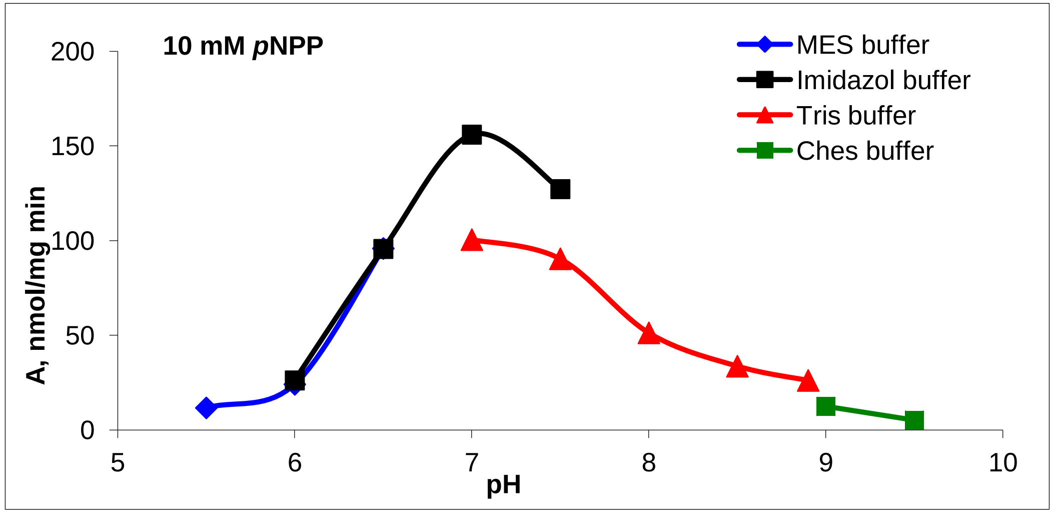

Fig. S7 pH-dependence of Ht-YitU$_{Bs}$ phosphatase activity towards GMP (**a**) and *p*NPP (**b**) in various buffers.

a

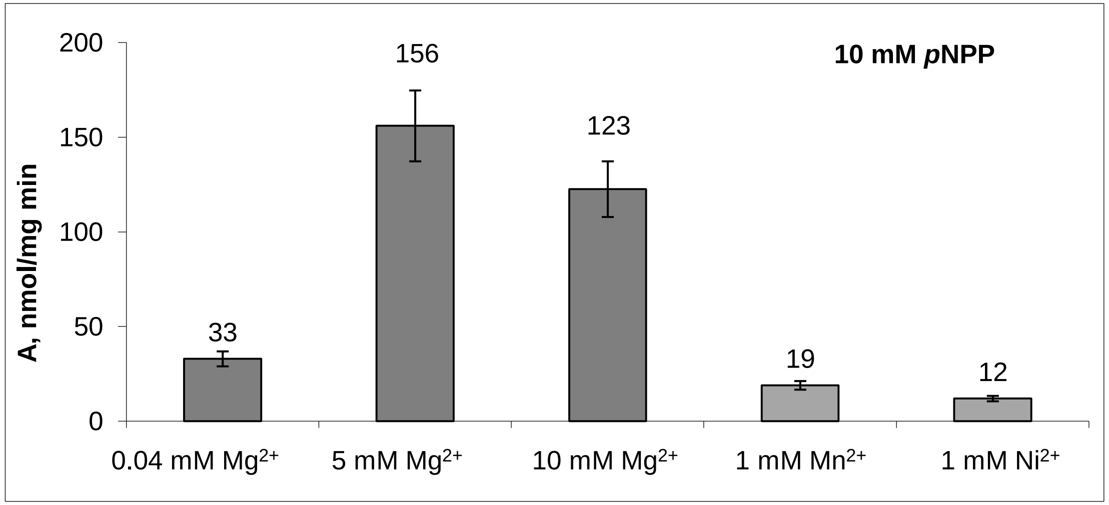

b

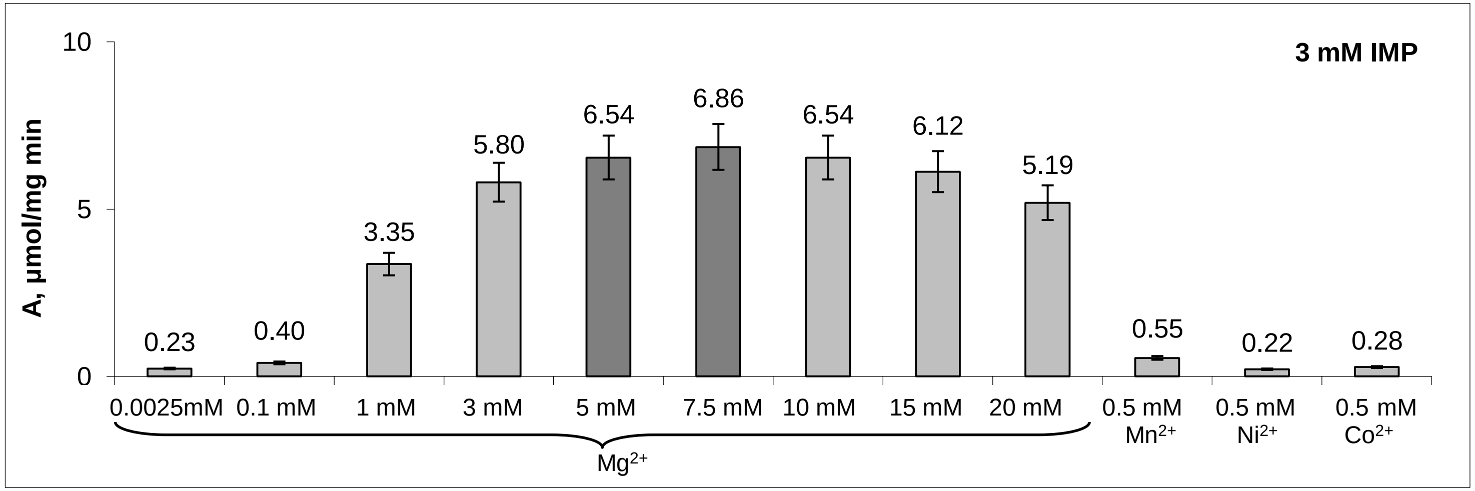

Fig. S8 Activation of Ht-YitU$_{Bs}$ by divalent cations in various concentrations. Ht-YitU$_{Bs}$ phosphatase activity towards *p*NPP (**a**) and IMP (**b**). The mean values of three independent experiments are shown; error bars indicate standard deviations

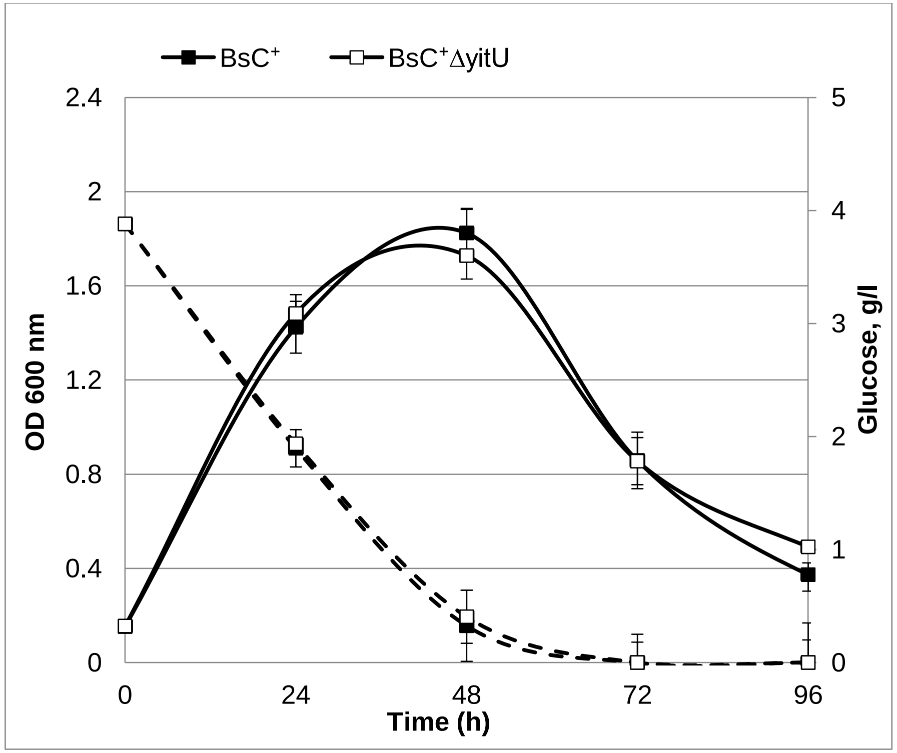

Fig. S9 Effect of *yitU* deletion on cell growth (solid lines) and glucose consumption (dashed lines) of BsC^+ (*B. subtilis* 168 *trpC*$^+$) during the cultivation in shaking flasks. The values are the means ± standard deviations of three independent experiments. Some error bars are smaller than the data point icons

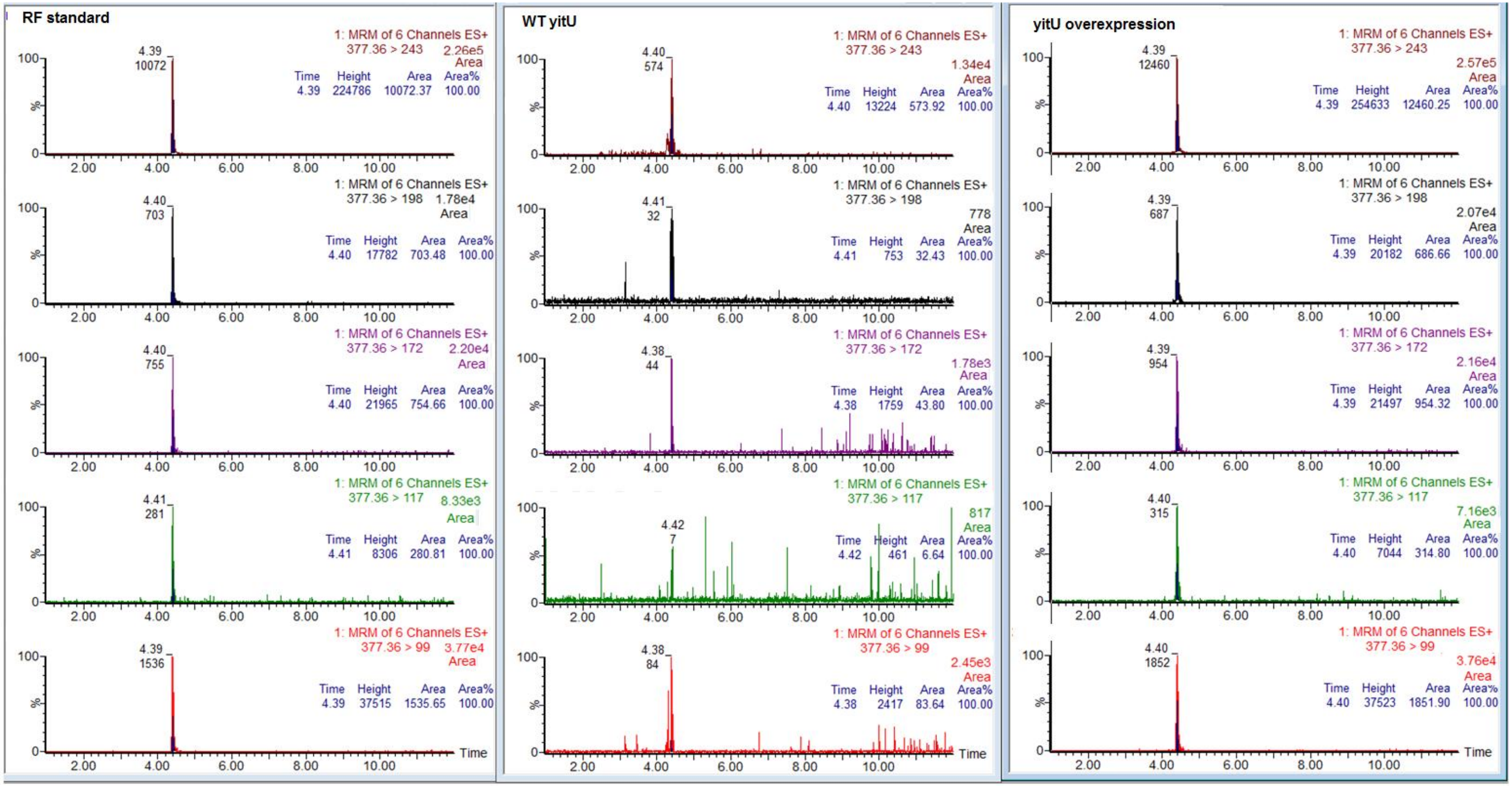

Fig. S10 LC-MS/MS chromatograms at transitions 377→243, 377→198, 377→172, 377→117 and 377→99 for standard RF solution of 0.45 mg/l (**RF standard**) and 10 times diluted cell-free culture supernatants of BsC^{+} (pMWAL1) (**WT yitU**) and BsC^{+} (pMWAL1-PyitU_{Ba}-yitU_{Bs}) (**yitU overexpression**)

a **b**

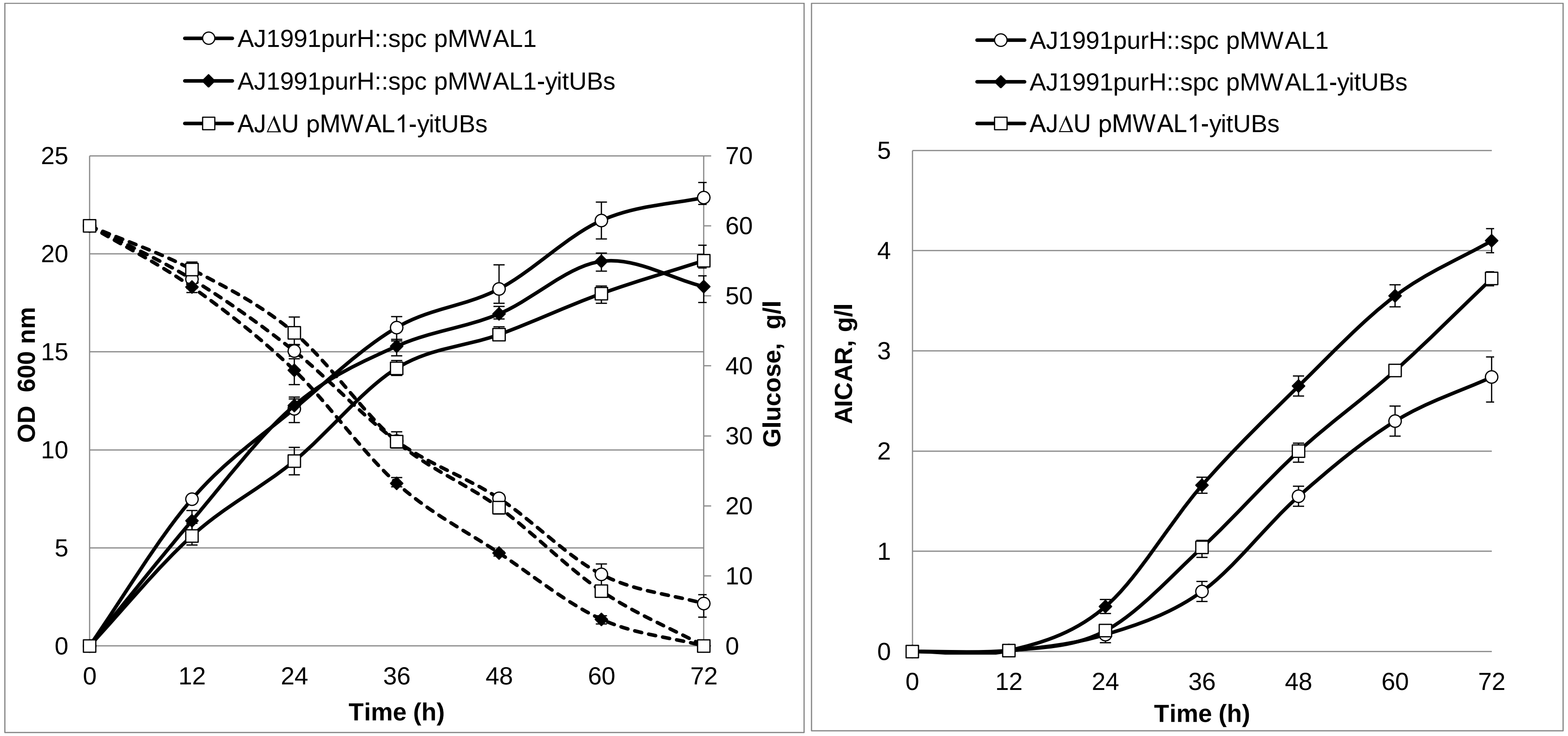

Fig. S11 Influence of *yitU*$_{Bs}$ overexpression on cell growth, glucose consumption **(a)** and AICAR production (**b**) in the *B. amyloliquefaciens* AICAR-producing strains AJ1991purH::spc and AJΔU. Solid lines indicate growth (**a**) and AICAR accumulation (**b**), while dashed lines indicate glucose consumption (**a**). The values are the means ± standard deviations of three independent experiments. Some error bars are smaller than the data point icons

References

Nicolas P, Mäder U, Dervyn E, Rochat T, Leduc A, Pigeonneau N, et al. (2012) Condition-dependent transcriptome reveals high-level regulatory architecture in *Bacillus subtilis*. Science 335(6072):1103–1106. https://doi.org/10.1126/science

Ravikumar V, Nalpas NC, Anselm V, Krug K, Lenuzzi M, Šestak MS, Domazet-Lošo T, Mijakovic I, Macek B (2018) In-depth analysis of *Bacillus subtilis* proteome identifies new ORFs and traces the evolutionary history of modified proteins. Sci Rep 8(1):17246. doi: 10.1038/s41598-018-35589-9
